# A Humidity-Powered Soft Robot with Fast Rolling Locomotion

**DOI:** 10.34133/2022/9832901

**Published:** 2022-05-14

**Authors:** Lei Fu, Weiqiang Zhao, Jiayao Ma, Mingyuan Yang, Xinmeng Liu, Lei Zhang, Yan Chen

**Affiliations:** ^1^School of Mechanical Engineering, Tianjin University, Tianjin 300350, China; ^2^Key Laboratory of Mechanism Theory and Equipment Design of Ministry of Education, Tianjin University, Tianjin 300350, China; ^3^Department of Biochemical Engineering, School of Chemical Engineering and Technology, Tianjin University, Tianjin 300350, China; ^4^Frontier Science Center for Synthetic Biology and Key Laboratory of Systems Bioengineering (MOE), Tianjin University, Tianjin 300350, China

## Abstract

A range of soft robotic systems have recently been developed that use soft, flexible materials and respond to environmental stimulus. The greatest challenge in their design is the integration of the actuator, energy sources, and body of robots while achieving fast locomotion and well-defined programmable trajectories. This work presents such a design that operates under constant conditions without the need for an externally modulated stimulus. By using a humidity-sensitive agarose film and overcoming the isotropic and random bending of the film, the robot, which we call the *Hydrollbot*, harnesses energy from evaporation for spontaneous and continuous fast self-rolling locomotion with a programmable trajectory in a constant-humidity environment. Moreover, the geometric parameters of the film were fine-tuned to maximize the rolling speed, and the optimised hydrollbot is capable of carrying a payload up to 100% of its own weight. The ability to self-propel fast under constant conditions with programmable trajectories will confer practical advantages to this robot in the applications for sensors, medical robots, actuation, etc.

## 1. Introduction

Many kinds of artificial materials have been developed to respond to light [1–7], heat [8–11], chemical substances [12, 13], magnetic fields [14–19], humidity [20–25], and other stimuli. They are highly promising for the development of intelligent and autonomous instruments such as actuators, sensors, or robots. However, a great challenge in the application of robots, especially soft robots [26, 27], is that in most of the cases the corresponding external stimuli require artificially controlled conditions to achieve repeated and controllable motion [28–30]. Apparently, the constant stimuli without external control make the robotic system much simpler constructed, less energy consuming, and thus easier to be applied in the real engineering practice. Currently, only a few reported soft robots can perform such motion under constant external stimuli [9, 20, 22, 31, 32]. For example, an elaborately designed bilayer self-locomotive ratcheted actuator performing straight motions was reported [22] that could be powered by either periodic changes or constant in environmental humidity. While the optimized speed of 0.24 body lengths per second (BL/s) could be achieved in periodic changed humidity, only a maximum speed of 0.0037 BL/s could be reached when placed on a moist surface with constant humidity. Chen et al. proposed a much faster actuator using a simple PPA polymer film with an intrinsic anisotropic structure driven by moisture [20]. The actuator could be programmed to perform direct bending and left-handed or right-handed helical motions. Besides humidity, devices driven by other stimuli have also been widely studied. Zhang et al. designed a bilayer photosensitive tubular motor that could be powered by light illumination and roll fast and straight away from light source [6]. Moreover, Wang et al. devised a self-propelled thermo-mechano-electrical actuation system that can perform perpetual and multimodal mobility [9]. Kotikian et al. 3D-printed soft robotic matter composed of liquid crystal elastomer (LCE) bilayers to form active hinges that interconnect polymeric tiles; based on which they created passively controlled, untethered soft robotic matter that adopts task-specific configurations responding to thermal stimuli [32]. One of their 3D-printed structures is a self-propelling column to move by rolling. Due to its higher motion efficiency, better environmental adaptability, and better stability, rolling motion has been taken as one of the advanced motion strategies in soft robotics. The slender tubes made of smart materials (such as LCE) bend to one side under external stimuli (such as heat or light), which results in asymmetric deformation and rolling [33, 34]. It is still on open question to achieve the rolling motion without forming the closed column or tube with soft materials. Therefore, the design challenge is to get a soft robot with a high locomotion speed (comparable to the speed of rigid robots which is 1 BL/s) [22] and predefined trajectories (straight, curved, etc.) under a constant stimulus.

To address this challenge, we focus on humidity-responsive materials, which can convert the energy from ambient evaporation into mechanical energy and thus have attracted increasing attentions to be used in humidity-driven actuators. Such materials include polymer gels [35–37], elastomers [38], shape memory polymers [39, 40], carbon nanomaterials [41], and electroactive polymers [42]. For application as actuators, humidity-responsive materials must provide fast water absorption and desorption. Here, we select a single layer of agarose film—a common and readily available biomacromolecule that meets these criteria [43]—as the main body and actuator of a soft robot. In general, such isotropic stimuli-responsive materials generate random and uncontrollable bending deformation. Hence, special structural design is developed to make this soft robot perform a regular and periodic rolling locomotion on a humid substrate; therefore, we call it the *Hydrollbot*. Meanwhile, we experimentally optimize the geometric design and adjust humidity to achieve the maximum rolling speed with programmed trajectories.

## 2. Results

### 2.1. Design of the Hydrollbot

**Figure 1:**
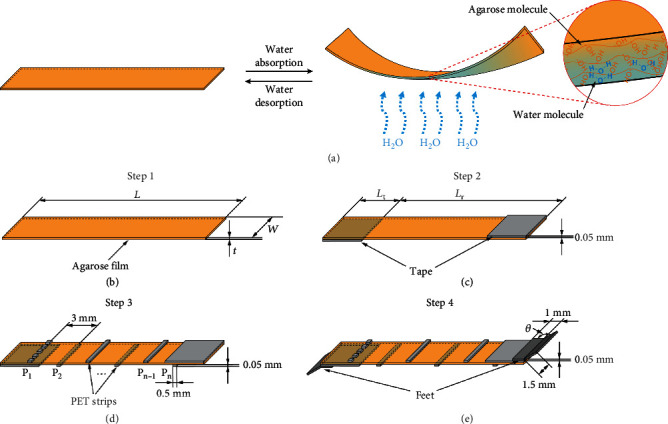
Design of the Hydrollbot. (a) Schematic illustration of the reversible bending and recovery of the humid-responsive single layer agarose film. (b–e) Construction process of the soft robot, Hydrollbot. (b) Agarose film with length, *L*, width, *w*, and thickness, *t*. (c) Two 0.05 mm thick tapes attached to the film on its bottom-left and top-right position with length, *L*_t_, to obtain the valid humidity-response length on each side, *L*_r_ = *L* − *L*_t_. (d) 0.5 mm wide and 0.05 mm thick polyethylene terephthalate (PET) strips attached to the agarose film with a 3 mm pitch alternatively on the top and bottom sides of the agarose film. (e) Two V-shaped PET feet attached to the tape with lengths of 1 and 1.5 mm, thicknesses of 0.05 mm, and variable angles, *θ*.

Agarose film contains abundant hydroxyl groups and is therefore very sensitive to moisture. It can rapidly absorb water under high humidity and desorb water under low humidity. As a result, it undergoes deformation and can aid in motion in very small humidity gradients (Figure 1(a)). Meanwhile, the experiment showed that the film could undergo over 1000 cycles of hydration and dehydration without saturation (Note S1). Hence, we used just one single layer of agarose film as the main body and actuator of the Hydrollbot (Figure 1(b)).

To create continuous rolling locomotion in one direction, we designed the robot with rotational symmetry about the middle line of its body. First, non-humidity-responsive tapes were manually attached to the film on the bottom-left and top-right ends to reduce the length of the responsive film (Figure 1(c)); as a result, asymmetric bending occurs along the film to produce the rolling motion. Second, to avoid the random twisting of the isotropic film, polyethylene terephthalate (PET) strips were attached to both sides of the film (Figure 1(d)). Third, two V-shaped PET sheets were attached to the tape layers to function as the feet of the robot, increasing friction and avoiding the possibility of reverse movement (Figure 1(e)). The dimensions of the robot are presented in Figures 1(b)–1(e). For a film length (*L*) of 18 mm (tape length, *L*_t_ = 3 mm), width (*w*) of 5 mm, and thickness (*t*) of 10 *μ*m, the total weight of the robot is 6.8 mg (Movie S1).

### 2.2. Rolling Locomotion

The Hydrollbot is designed to transfer the bending motion of the film into rolling locomotion in a predefined direction. To set up the locomotion experiments, a piece of filter paper was placed flat on the vessel filled with water to work as the humid substrate for robots to move. The humidity was measured by a humidity sensor put on the substrate (Note S2).

**Figure 2:**
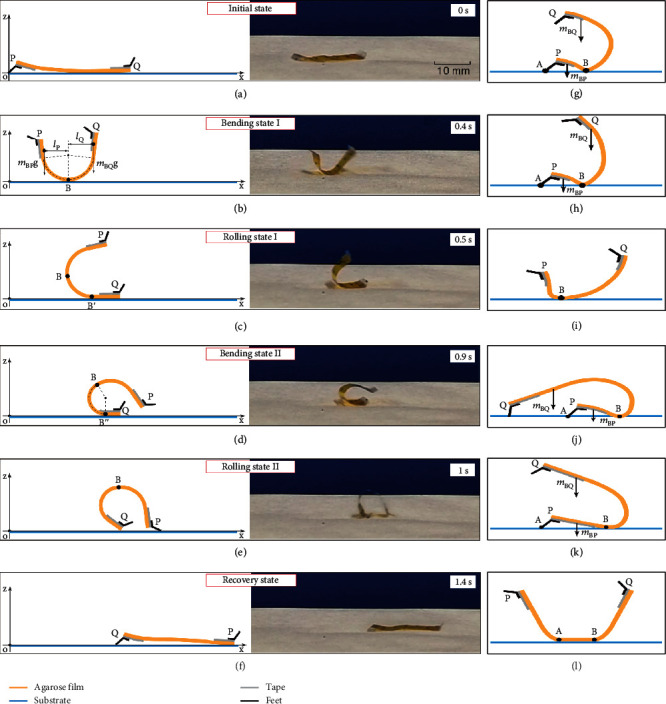
Rolling process of the Hydrollbot. (a) Initial state. The robot is in flat configuration with the two ends labeled as P and Q. (b) Bending state I. The black point (labeled as B) is the contact point between the robot film and substrate. *m*_BQ_ and *m*_BP_ represent the mass of BQ and BP parts, respectively. *l*_p_ and *l*_Q_ represent the mass center position of BQ and BP parts, respectively. (c) Rolling state I. The robot rolls to right with the contact point moves to B′. (d) Bending state II. Further bending at Q end and unbending at P end with contact point at B^″^. (e) Rolling state II. Rolling about foot Q until foot P reaches the substrate. (f) Recovery state of this cycle as the robot returns to flat configuration, which is also the initial state of the next rolling cycle. The corresponding video is shown in Movie S2. Here, design parameters of the robot on the right part of (a)–(f) are film length (*L*) of 18 mm (tape length, *L*_t_ = 3 mm), width (*w*) of 5 mm, thickness (*t*) of 10 *μ*m, and weight of 6.8 mg. (g–i) The role of the PET feet in preventing reverse rolling. (g) Reverse rolling state of the Hydrollbot when the left foot touches the substrate. Point A represents the support point between the left foot and the substrate. (h) Unbending state of the robot when reverse rolling is prevented. (i) Rolling state similar to that of the robot in (c). (j, k) The role of the tape length, *L*_t_, in preventing reverse rolling. (j) Reverse rolling state with a long film, in which reverse rolling cannot be prevented. (k) Reverse rolling state with a longer tape where the reverse rolling can be prevented. (l) Too long film will make two ends bend up while the middle part of the film is straight, which then increases the duration of the rolling cycle.

A typical rolling process of Hydrollbot is shown in Figure 2 and Movie S2. When the robot in the initial state (Figure 2(a)) is placed on the substrate, the lower side of the film absorbs water molecules from the substrate to produce bending deformation. Because end P of the film is covered by tape and lifted up by the PET feet, end Q is closer to the moist surface than end P; hence, the bending speed at film BQ is faster, causing the overall center of gravity to be between BQ, whereas B is the contact point between robot film and substrate. As shown in Figure 2(b), (*m*_BQ_ g) · *l*_Q_ > (*m*_BP_ g) · *l*_P_, which causes the robot to roll to the end Q (Figures 2(b) and 2(c)). When the robot enters rolling state I (Figure 2(c)), the film near the substrate absorbs moisture and deforms into bending state II (Figure 2(d)). The gravity center of the robot moves to the right side of the contact point B′, causing the robot to tumble clockwise. After the robot completes the tumbling motion, it enters rolling state II (Figure 2(e)), whereby the lower side of the film begins to absorb water molecules and the upper side begins to lose water molecules, which causes the robot to deform into the recovery state illustrated (Figure 2(f)). The recovery state is similar to the initial state (Figure 2(a)), and the robot then starts to deform again into bending state I. Hence, we define the time required for the rolling processes shown in Figures 2(a)–2(f) as one rolling cycle, which has a duration of 1.4 s averagely with error ± 0.2 s for the robot sample shown in Figure 2. Considering that the maximum displacement in one cycle is equal to the length of the robot, the speed under ideal rolling performance would be 0.714 BL/s.

The prolonged cycle is due to the tendency of reverse rolling caused by the nonuniform bending or high humidity. In this case, the fast bending speed of the Q side causes the center of gravity to move towards P (Figure 2(g)). Thus, the robot will roll counterclockwise, and end P will touch the substrate. Then, the center of gravity is between supporting points A and B, which prevents further rolling. At the same time, film BQ is far from the humid substrate, and the moisture is partially blocked by film PB, so film BQ will recover to a flat state, and its center of gravity will move to the right (Figure 2(h)). As the result, the robot rolls clockwise into bending state I (Figure 2(i)), which is similar to the state in Figure 2(b). The subsequent locomotion is also the same as that in Figures 2(c)–2(f). Therefore, the feet play a critical role in preventing reverse rolling, which is largely influenced by the foot angle *θ* illustrated in Figure 1(e). We chose 120° as the optimized angle through a number of experiments of robots with *θ* = 90°, 120°, and 150°. It is observed that 120° feet provide better support when the robot tends to roll reversely than 90° and without hindering forward rolling while the 150° feet do (Note S3).

Meanwhile, reverse rolling can also be caused by a large robot length, especially the large active length (*L*_r_ = *L*–*L*_t_), which the film length uncovered by the tapes. For *t* = 10 *μ*m and RH ≈ 60%, when *L* ≤ 18 mm with *L*_t_ = 3 mm, we observed no reverse rolling. When *L* = 21 mm still with *L*_t_ = 3 mm, the probability of reverse rolling increased to 25% (Note S3). As shown in Figure 2(j), if the film length is too long, the center of gravity will move to the left of support point A, which may lead to reverse rolling. By increasing the tape length *L*_t_, we can increase the distance between points A and B and keep the center of gravity on the right of point A (Figure 2(k)), and then, no reverse rolling will occur. When *L*_t_ ≥ 4.5 mm with *L* = 21 mm, reverse rolling never occurs.

The locomotion speed of the Hydrollbot is affected by both the geometric parameters of the robot (including the film length, tape length, and film thickness), the substrate humidity, and the material properties.

**Figure 3:**
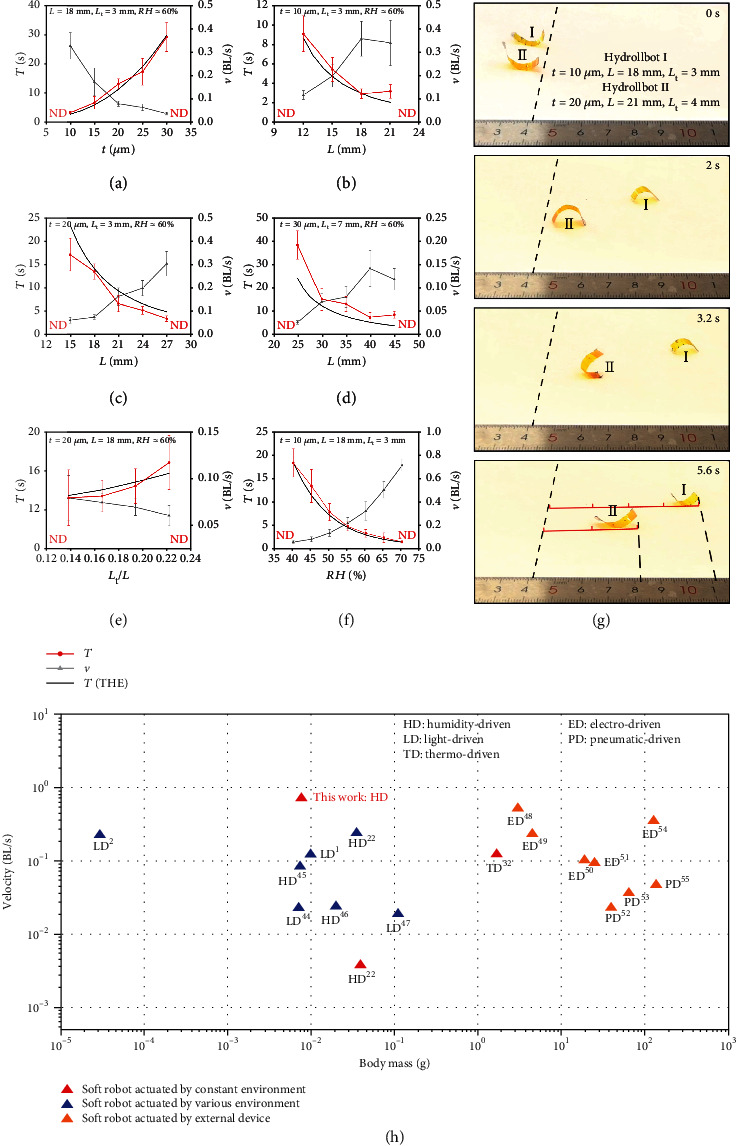
Parametric study of structural parameters, environmental humidity on the locomotion of the Hydrollbot. (a) When *L* = 18 mm, *L*_t_ = 3 mm, and RH ≈ 60%, locomotion cycle *T* and velocity *vvs.* agarose film thickness, *t*. (b) When *t* = 10 *μ*m, *L*_t_ = 3 mm, and RH ≈ 60%, *T* and *vvs.L*. (c) When *t* = 20 *μ*m, *L*_t_ = 3 mm, and RH ≈ 60%, *T* and *vvs.L*. (d) When *t* = 30 *μ*m, *L*_t_ = 7 mm, and RH ≈ 60%, *T* and *vvs.L*. (e) When *t* = 20 *μ*m, *L* = 18 mm, and RH ≈ 60%, *T* and *vvs.L*_t_/*L*. (f) When *t* = 10 *μ*m, *L* = 18 mm, and *L*_t_ = 3 mm, *T* and *vvs.*RH. (g) Locomotion comparison between Hydrollbot I (*t* = 10 *μ*m, *L* = 18 mm, and *L*_t_ = 3 mm) and Hydrollbot II (*t* = 20 *μ*m, *L* = 21 mm, and *L*_t_ = 4 mm) at RH ≈ 70%. (h) Velocity of soft robots measured in body length per second vs. body mass [44–55]. In (a)–(f), error bars represent s.d. of at least four measurements; the red lines for locomotion cycle time, *T*, in experiments; the grey lines for velocity, *v*, in experiments; and the black lines for the cycle time calculated from the setup analytical model.

First, the speed is highly sensitive to the film thickness. We kept *L* = 18 mm, *L*_t_ = 3 mm, and RH ≈ 60% and varied *t* from 10 to 30 *μ*m. The cycle duration, *T*, and robot speed, *v* ( = *L*/*T*), were measured for continuous 4-8 cycles, which was decided by the robot length and substrate length. The duration of the rolling cycle increased with film thickness (Figure 3(a)) because a thicker film must absorb more water molecules than a thinner film to produce a similar curvature. Under the same conditions, the speed of 10 *μ*m robot is nearly 10 times of 30 *μ*m robot.

Second, consider the film length *L* and tape length *L*_t_. For a film of *t* = 10 *μ*m and RH ≈ 60%, we fixed *L*_t_ = 3 mm and varied *L* from 12 to 21 mm. Bending state I of the Hydrollbot in Figure 2(b) shows that the robot rolls when the middle section of the film becomes approximately semicircular and the bending speed of the film is constant for a given humidity. As *L*_r_ increases, the film more rapidly approaches a semicircular shape. However, if *L*_r_ approaches 18 mm, two ends bend up while the middle part of the film is straight (see Figure 2(l)), which then increases the duration of the rolling cycle. Meanwhile, the robot experiences no rolling when it is too short and reverse rolling when it is too long. Hence, suitable *L* is between 12 and 21 mm. Its lower limit can be further reduced by increasing the humidity, reducing the film thickness, or reducing the tape length. The results showed that *T* decreases and *v* increases as *L* increases (Figure 3(b)). This trend is identical for all film thickness we tested between 10 *μ*m and 30 *μ*m, while Figure 3(c) for *t* = 20 *μ*m, *L*_t_ = 3 mm, and *L* between 15 and 27 mm, and Figure 3(d) for *t* = 30 *μ*m, *L*_t_ = 7 mm, and *L* between 25 and 45 mm. In fact, it is the active length *L*_r_ = *L* − *L*_t_ which effects the robot speed and cycle. We fix *L* and vary *L*_t_ to find the optimized geometry. When *L*_t_ is too long, the robot cannot roll with two unbend long ends, and when *L*_t_ is too short, at bending state I, the left and right parts are almost with the same weight and B is too close to the robot center to make the robot balance at this state without further rolling. After studying all the experiments, we find that ideal *L*_t_/*L* is between 0.111 and 0.278. For example, for *t* = 20 *μ*m and RH ≈ 60%, set *L* = 18 mm, we changed *L*_t_ from 2.5 to 4 mm, which leads to the decrease of *v* and increase of *T* (Figure 3(e)).

Third, apart from the dimensional parameters of the Hydrollbot, humidity strongly influences the locomotion. To investigate the effect of humidity, we set *L* = 18 mm, *L*_t_ = 3 mm, and *t* = 10 *μ*m and changed RH from 40% to 70% (±2%) by adjusting the water temperature between 23 and 61°C in the vessel. Note that the humidity could only be controlled within a range of ±2%. The effect of humidity on the rolling locomotion is shown in Figure 3(f). Because the film absorbs water molecules more rapidly at higher humidity, the bending speed is faster than at lower humidity, which increases the rolling speed and decreases the duration of the rolling cycle. Thus, a higher humidity gives a faster rolling speed. The same robot moves 12 times faster on the RH-70% substrate than RH-40% one. It should be noted that if the film is placed in a substrate with humidity higher than 70% (±2%), it will be severely deformed, and reverse rolling is likely to occur. We also tested the lowest humidity required to achieve steady rolling locomotion, which was 40%; at lower humidity, the robot cannot bend enough, hence only tumble left and right without performing rolling motion. If the values of *t*, *L*, and/or *L*_t_ are changed, the humidity limits will change accordingly. For example, when *t* is 30 *μ*m, *L* = 35 mm, and *L*_t_ = 7 mm, the ideal RH is in the range of 33% to 70% with 70% humidity offering the highest speed. All the experiment data for Figures 3(a)–3(f) can be found in Note S4.

Fourth, to find out the effect of material property on the locomotion speed, we built a simplified analytical model to estimate the locomotion speed of the robot using energy method based on experimental observation. It was assumed in the model that the robot absorbed energy from the humid environment at a constant rate, which was used to bend the robot and overcome its gravity. Then, by equating the humidity energy absorption of the robot to the summation of bending energy gain and gravitational potential energy variation, the locomotion cycle was derived (Note S5). It should be pointed out that as a type of natural polysaccharide prepared from agar, the structure and properties of the agarose polymers may be notably influenced by the raw materials; consequently, the mechanical properties of the films may also be affected by the products from different vendors and batches.

The analytical results drawn as black lines in Figures 3(a)–3(f) are found to match the experimental data reasonably well. Both the experiments and analytical model indicate that the locomotion speed increases with film length and humidity, while it reduces with film thickness and tape length, according to which we can maximize the locomotion speed of the robot. When *t* = 10 *μ*m, *L* = 18 mm, *L*_t_ = 3 mm, and RH = 70%, the maximum speed reaches 0.714 BL/s. The locomotion comparison between this optimized robot and the one with randomly selected parameters (*t* = 20 *μ*m, *L* = 21 mm, *L*_t_ = 4 mm) is presented in Figure 3(g) (Movie S3). Meanwhile, we compared the locomotion performance of the Hydrollbot with other soft robots with the velocity measured in body length (BL) per second and body mass (Figure 3(h)). As the optimized Hydrollbot has velocity of 0.714 BL/s and weight of 6.8 mg, it is much faster than most soft robots actuated by various environments and two magnitudes faster than current reported state-of-the-art humidity-responsive soft robots that move in constant environments, which was 0.0037 BL/s of the similar weight in [22]. The soft robots actuated by the external devices have a larger weight but lower speed than the Hydrollbot. While the rigid robots and animals are of a speed above 1 BL/s, our Hydrollbot has the speed close to this lower limit.

Meanwhile, the loading-carrying capability of Hydrollbot has been demonstrated in Movie S4, in which the extra loading was put on the position of two feet without disturbing the asymmetric weight distribution so as to keep the rolling motion. From the experiments in Note S6, we can tell that the proposed Hydrollbot is capable of carrying a payload up to 100% of its own weight.

**Figure 4:**
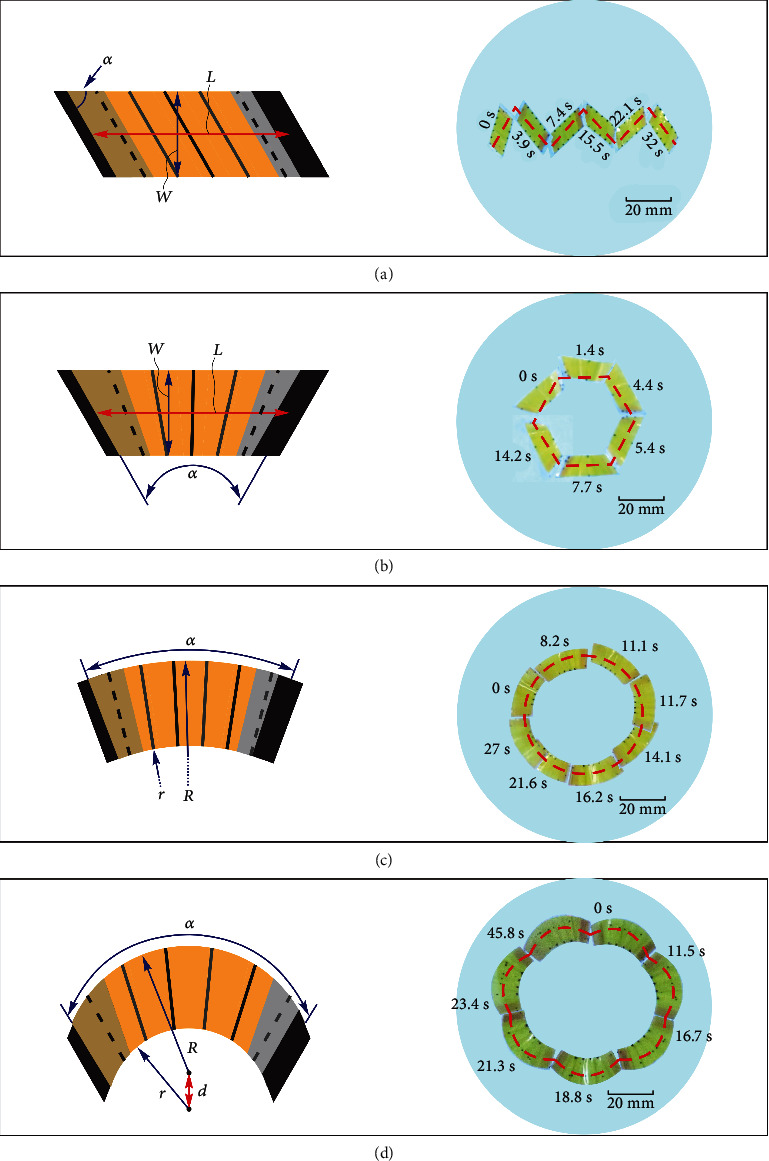
Programmable trajectories of the Hydrollbots. (a–d) Hydrollbots with zigzag, polygonal, circular, and flower-shaped trajectories, respectively. Experimental observations for (a) zigzag, (b) hexagonal, (c) circular, and (d) flower-shaped trajectories. The geometric parameters for different trajectories were as follows: zigzag (*L* = 18 mm, *w* = 8 mm, and *α* = 60°), hexagonal (*L* = 18 mm, *w* = 8 mm, and *α* = 60°), circular (*R* = 26 mm, *r* = 18 mm, and *α* = 50°), and flower-shaped circular (*R* = 21 mm, *r* = 11 mm, *α* = 60°, and *d* = 4 mm) with all film thickness *t* = 10 *μ*m and humidity RH ≈ 60%.

A unique feature of Hydrollbot is that its trajectory can be programmed simply by changing the robot geometry. Because the agarose film is isotropic, we can change the rolling direction by designing the shape of the film and the positions of the feet attached to its short sides; additionally, we can control the bending direction of the film by adjusting the relative positions of the PET strips. Four representative example geometries and their corresponding trajectories are shown in Figures 4(a)–4(d) (Movie S5). When the film shape is a parallelogram and the PET strips are parallel to the two short sides (Figure 4(a)), the Hydrollbot moves in a zigzag trajectory. A trapezoidal film with PET strips equally divided between the two oblique sides (Figure 4(b)) can achieve a polygonal trajectory. The size of the polygonal trajectory can be changed by altering the median line length of the trapezoid, and the shape of the polygonal trajectory can be varied by adjusting the angles of the trapezoid. Furthermore, the Hydrollbot in Figure 4(c) uses sectorized film; the PET strips equally divide the sector to generate a circular trajectory with a diameter that can be varied by changing the middle diameters of the sectors. We can also generate a flower-shaped trajectory by changing the film formed by two arches with different centers and radii (Figure 4(d)). These programmable characteristics allow the Hydrollbot to perform complex trajectories, which are essential for practical applications.

## 3. Conclusions

Our humidity-responsive soft robot can roll on a humid surface without any additional energy input. Unlike existing robots of this kind, which require an environment with actively controlled variable humidity to move, the rolling locomotion of the Hydrollbot is powered by the inherent difference in humidity between two sides of the robot body when operating on a constant-humidity substrate. The optimized rolling speed is 0.714 BL/s, which is two magnitudes faster than current reported state-of-the-art humidity-responsive soft robots that move in constant environments. Another advantage of the Hydrollbot is that in addition to performing straight-line locomotion, it can be programmed to move in curved, zigzag, or other predefined trajectories by simply altering its geometry. We expect that these features will greatly advance the applications of humidity-driven soft robots in medical treatment, sensing, actuation, and so on. Future research will focus on the design of more advanced Hydrollbot structures that can autonomously perform complex locomotion paths. Moreover, incorporation of new materials may enable the Hydrollbot responsive to multiple stimuli and with more intelligent and reliable performance.

## 4. Materials and Methods

### 4.1. Preparation of the Agarose Films

Agarose films were prepared according to the following procedure [43]. 6.0 g of agarose powder was added to 180 ml of dimethylformamide with vigorous stirring, and the mixture was heated to 95°C to completely dissolve the agarose powder. Then, 0.12 g of phenol red was added to the solution as dye to stain the product. Next, the solution was cast onto glass molds (18 cm × 18 cm) and dried under ambient conditions for 3 days to form the films. The film thickness was tuned by the amount of solution added to the mold and measured with a micrometer caliper.

### 4.2. Preparation of the Hydrollbot

Firstly, agarose film was cut into rectangle by a laser cutter. The width of the rectangle was fixed to 5 mm to avoid sideway rolling, while the length was tuned to achieve optimized performance. Then, tapes with specific size were attached to the two ends of the film. As shown in Figure 1(c), the tape at the left end was attached manually to the lower side of the film, and the tape at the right end was attached to the upper side of the film. Then, 0.05 mm thick PET film was cut into 0.5 mm wide and 5 mm long strips and attached manually to the film (Figure 1(d)), and for a thin film (*t* ≤ 20 *μ*m), it is not necessary to attach the PET strips on the area with tapes, which is P_1_ and P*_n_* marked as dash line on Figure 1(d). Two adjacent PET strips were 3 mm apart and attached to different sides of the film alternatively. Lastly, two feet made by PET film were adhered to the two ends of the film (Figure 1(e)). In the robot in Figure 2, the film weighs 1.8 mg, the two tapes weigh 1.5 mg, the three PET strips weigh 0.9 mg, and the two feet weigh 2.6 mg; hence, the total weight of the robot is 6.8 mg.

To prepare robot with zigzag trajectory, the film was cut into parallelogram and only 3 PET strips were attached parallel to the two short sides. To prepare robot with polygonal trajectory, the film was cut into trapezoid and 3 PET strips equally divided the angle between the two oblique sides. To prepare robot with circular trajectory, the film shape was changed into sector and 4 PET strips equally divided the sector angle. The robot with flower-shaped trajectory was developed by changing the film formed by two arches with different centers and radii. 4 PET strips equally divided the angle between the two short sides. Because the film thickness *t* = 10 *μ*m, the PET strips allocated on the area of tapes were not attached, which is demonstrated as dash line in Figure 4.

### 4.3. Experimental Details

To evaluate the rolling properties of the robot, Hydrollbots of different sizes were placed on a filter paper (*φ*15, GB/T1914-2007) above a vessel (1 liter) in an oil bath (Figure S2). The water volume in the vessel is 800 ml, and the distance between the water surface and the substrate was 60 mm. The humidity was adjusted by changing temperature (23°C~61°C) in the vessel. Humidity sensor (±2% RH, Anymetre TH21E, China) was placed on the substrate to measure the humidity.

## Data Availability

Data supporting the findings of this study are available in the main text or the supplementary information.

## Supplementary Materials

Supplementary Materials Note S1: materials. Note S2: setup of the locomotion experiments of Hydrollbots. Note S3: mechanisms to prevent undesirable rolling. Note S4: the lists of data in Figure 3. Note S5: locomotion speed of Hydrollbots with different structural parameters. Note S6: the loading-carrying capability of the Hydrollbot. Note S7: the optimization on the width of PET strips and the distance between adjacent strips of the Hydrollbot. Figure S1: the cyclic hydration and dehydration of the agarose film. Figure S2: the experimental setup for the locomotion of Hydrollbots. Figure S3: the analytical model of the rolling process of the Hydrollbot. Figure S4: the curve fitting result of the time *T*__1_ in four different conditions with energy absorption rate *ξ*. Figure S5: the states before (a) and after (b) bouncing of the Hydrollbot. Figure S6: the rolling comparison of two Hydrollbots with different structure parameters. Figure S7: design of the Hydrollbots (a) without payload and (b) with payload. Figure S8: the effect of the width and pitch of the PET strips on the rolling locomotion. Movie S1: preparation of the Hydrollbot. Movie S2: rolling process of the Hydrollbot. Movie S3: rolling comparison of Hydrollbots with different structural parameters. Movie S4: rolling process of the Hydrollbots with different weights. Movie S5: programmable trajectories of the Hydrollbots. Movie S6: cyclic hydration and dehydration of the agarose film. Movie S7: the effect of the width and pitch of the PET strips on the rolling locomotion.

## References

[1] Ji M., Jiang N., Chang J., Sun J. (2014). Near-infrared light-driven, highly efficient bilayer actuators based on polydopamine-modified reduced graphene oxide. *Advanced Functional Materials*.

[2] Lv J.-A., Liu Y., Wei J., Chen E., Qin L., Yu Y. (2016). Photocontrol of fluid slugs in liquid crystal polymer microactuators. *Nature*.

[3] Li C., Iscen A., Sai H. (2020). Supramolecular-covalent hybrid polymers for light-activated mechanical actuation. *Nature Materials*.

[4] Palagi S., Mark A. G., Reigh S. Y. (2016). Structured light enables biomimetic swimming and versatile locomotion of photoresponsive soft microrobots. *Nature Materials*.

[5] Iamsaard S., Aßhoff S. J., Matt B. (2014). Conversion of light into macroscopic helical motion. *Nature Chemistry*.

[6] Zhang X., Yu Z., Wang C. (2014). Photoactuators and motors based on carbon nanotubes with selective chirality distributions. *Communications*.

[7] Li C., Iscen A., Palmer L. C., Schatz G. C., Stupp S. I. (2020). Light-driven expansion of spiropyran hydrogels. *Journal of the American Chemical Society*.

[8] Yoshida R., Uchida K., Kaneko Y. (1995). Comb-type grafted hydrogels with rapid deswelling response to temperature changes. *Nature*.

[9] Wang X.-Q., Tan C. F., Chan K. H. (2018). In-built thermo-mechanical cooperative feedback mechanism for self-propelled multimodal locomotion and electricity generation. *Communications*.

[10] Straub A. P., Yip N. Y., Lin S., Lee J., Elimelech M. (2016). Harvesting low-grade heat energy using thermo-osmotic vapour transport through nanoporous membranes. *Energy*.

[11] Yang Y., Terentjev E. M., Zhang Y. (2019). Reprocessable thermoset soft actuators. *Angewandte Chemie International Edition*.

[12] Hu Z. B., Zhang X. M., Li Y. (1995). Synthesis and application of modulated polymer gels. *Science*.

[13] Techawanitchai P., Ebara M., Idota N., Asoh T. A., Kikuchi A., Aoyagi T. (2012). Photo-switchable control of pH-responsive actuators via pH jump reaction. *Soft Matter*.

[14] Hu W., Lum G. Z., Mastrangeli M., Sitti M. (2018). Small-scale soft-bodied robot with multimodal locomotion. *Nature*.

[15] Erb R. M., Martin J. J., Soheilian R., Pan C., Barber J. R. (2016). Actuating soft matter with magnetic torque. *Advanced Functional Materials*.

[16] Rahmer J., Stehning C., Gleich B. (2017). Spatially selective remote magnetic actuation of identical helical micromachines. *Science Robotics*.

[17] Kim J., Chung S. E., Choi S.-E., Lee H., Kim J., Kwon S. (2011). Programming magnetic anisotropy in polymeric microactuators. *Nature Materials*.

[18] Deng H., Sattari K., Xie Y., Liao P., Yan Z., Lin J. (2020). Laser reprogramming magnetic anisotropy in soft composites for reconfigurable 3D shaping. *Nature Communications*.

[19] Kim Y., Parada G. A., Liu S., Zhao X. (2019). Ferromagnetic soft continuum robots. *Robotics*.

[20] Chen Q., Yan X., Lu H., Zhang N., Ma M. (2019). Programmable polymer actuators perform continuous helical motions driven by moisture. *ACS Applied Materials & Interfaces*.

[21] Mu J., Wang G., Yan H. (2018). Molecular-channel driven actuator with considerations for multiple configurations and color switching. *Communications*.

[22] Shin B., Ha J., Lee M. (2018). Hygrobot: a self-locomotive ratcheted actuator powered by environmental humidity. *Robotics*.

[23] Ma M., Guo L., Anderson D. G., Langer R. (2013). Bio-inspired polymer composite actuator and generator driven by water gradients. *Science*.

[24] Arazoe H., Miyajima D., Akaike K. (2016). An autonomous actuator driven by fluctuations in ambient humidity. *Nature Materials*.

[25] Treml B. E., McKenzie R. N., Buskohl P. (2018). Autonomous motility of polymer films. *Advanced Materials*.

[26] Hwang G., Paula A. J., Hunter E. E. (2019). Catalytic antimicrobial robots for biofilm eradication. *Science Robotics*.

[27] Wu Y., Yim J. K., Liang J. (2019). Insect-scale fast moving and ultrarobust soft robot. *Robotics*.

[28] Sun Z., Yamauchi Y., Araoka F. (2018). An anisotropic hydrogel actuator enabling earthworm-like directed peristaltic crawling. *Angewandte Chemie International Edition*.

[29] Li J., Zhang R., Mou L. (2019). Photothermal bimorph actuators with in-built cooler for light mills, frequency switches, and soft robots. *Advanced Functional Materials*.

[30] Wang Z., Li K., He Q., Cai S. (2019). A light-powered ultralight tensegrity robot with high deformability and load capacity. *Advanced Materials*.

[31] Gelebart A. H., Jan Mulder D., Varga M. (2017). Making waves in a photoactive polymer film. *Nature*.

[32] Kotikian A., McMahan C., Davidson E. C. (2019). Untethered soft robotic matter with passive control of shape morphing and propulsion. *Robotics*.

[33] Ahn C., Li K., Cai S. (2018). Light or thermally powered autonomous rolling of an elastomer rod. *ACS Applied Materials & Interfaces*.

[34] Zhai F., Feng Y., Li Z. (2021). 4D-printed untethered self-propelling soft robot with tactile perception: rolling, racing, and exploring. *Matter*.

[35] Sun X.-C., Xia H., Xu X.-L., Lv C., Zhao Y. (2020). Ingenious humidity-powered micro-worm with asymmetric biped from single hydrogel. *Sensors and Actuators B: Chemical*.

[36] Chu S., Majumdar A. (2012). Opportunities and challenges for a sustainable energy future. *Nature*.

[37] Sidorenko A., Krupenkin T., Taylor A., Fratzl P., Aizenberg J. (2007). Reversible switching of hydrogel-actuated nanostructures into complex micropatterns. *Science*.

[38] Ohm C., Brehmer M., Zentel R. (2010). Liquid crystalline elastomers as actuators and sensors. *Advanced Materials*.

[39] Lendlein A., Jiang H. Y., Junger O., Langer R. (2005). Light-induced shape-memory polymers. *Nature*.

[40] Davis K. A., Burke K. A., Mather P. T., Henderson J. H. (2011). Dynamic cell behavior on shape memory polymer substrates. *Biomaterials*.

[41] Lima M. D., Li N., Jung de Andrade M. (2012). Electrically, chemically, and photonically powered torsional and tensile actuation of hybrid carbon nanotube yarn muscles. *Science*.

[42] Taccola S., Greco F., Sinibaldi E., Mondini A., Mazzolai B., Mattoli V. (2015). Toward a new generation of electrically controllable hygromorphic soft actuators. *Advanced Materials*.

[43] Zhang L., Liang H., Jacob J., Naumov P. (2015). Erratum: Photogated humidity-driven motility. *Communications*.

[44] Shahsavan H., Aghakhani A., Zeng H. (2020). Bioinspired underwater locomotion of light-driven liquid crystal gels. *PNAS*.

[45] Ma Y., Zhang Y., Wu B., Sun W., Li Z., Sun J. (2011). Polyelectrolyte multilayer films for building energetic walking devices. *Angewandte Chemie International Edition*.

[46] Lee S.-W., Prosser J. H., Purohit P. K., Lee D. (2013). Bioinspired hygromorphic actuator exhibiting controlled locomotion. *ACS Macro Letters*.

[47] Zhao Y., Xuan C., Qian X. (2019). Soft phototactic swimmer based on self-sustained hydrogel oscillator. *Science Robotics*.

[48] Huang X., Kumar K., Jawed M. K. (2018). Chasing biomimetic locomotion speeds: creating untethered soft robots with shape memory alloy actuators. *Robotics*.

[49] Shintake J., Cacucciolo V., Shea H., Floreano D. (2018). Soft biomimetic fish robot made of dielectric elastomer actuators. *Soft Robotics*.

[50] Zhang W., Guo S., Asaka K. (2007). Development of an underwater biomimetic microrobot with compact structure and flexible locomotion. *Microsystem Technologies*.

[51] Cao C., Diteesawat R. S., Rossiter J., Conn A. T. A reconfigurable crawling robot driven by electroactive artificial muscle.

[52] Tang Y., Zhang Q., Lin G., Yin J. (2018). Switchable adhesion actuator for amphibious climbing soft robot. *Soft Robotics*.

[53] Kwok S. W., Morin S. A., Mosadegh B. (2014). Magnetic assembly of soft robots with hard components. *Advanced Functional Materials*.

[54] Yang T., Xiao Y., Zhang Z. (2018). A soft artificial muscle driven robot with reinforcement learning. *Scientific Reports*.

[55] Robertson M. A., Paik J. (2017). New soft robots really suck: vacuum-powered systems empower diverse capabilities. *Robotics*.

