# Supplementary material for: A Humidity-Powered Soft Robot with Fast Rolling Locomotion
Source: Research (Wash D C). 2022 May 14;2022:9832901. doi: 10.34133/2022/9832901 (PMC9125428; doi:10.34133/2022/9832901)
Supplement: Supplementary Materials — Note S1: materials. Note S2: setup of the locomotion experiments of Hydrollbots. Note S3: mechanisms to prevent undesirable rolling. Note S4: the lists of data in Figure 3. Note S5: locomotion speed of Hydrollbots with different structural parameters. Note S6: the loading-carrying capability of the Hydrollbot. Note S7: the optimization on the width of PET strips and the distance between adjacent strips of the Hydrollbot. Figure S1: the cyclic hydration and dehydration of the agarose film. Figure S2: the experimental setup for the locomotion of Hydrollbots. Figure S3: the analytical model of the rolling process of the Hydrollbot. Figure S4: the curve fitting result of the time T_1 in four different conditions with energy absorption rate ξ. Figure S5: the states before (a) and after (b) bouncing of the Hydrollbot. Figure S6: the rolling comparison of two Hydrollbots with different structure parameters. Figure S7: design of the Hydrollbots (a) without payload and (b) with payload. Figure S8: the effect of the width and pitch of the PET strips on the rolling locomotion. Movie S1: preparation of the Hydrollbot. Movie S2: rolling process of the Hydrollbot. Movie S3: rolling comparison of Hydrollbots with different structural parameters. Movie S4: rolling process of the Hydrollbots with different weights. Movie S5: programmable trajectories of the Hydrollbots. Movie S6: cyclic hydration and dehydration of the agarose film. Movie S7: the effect of the width and pitch of the PET strips on the rolling locomotion. [file 9832901.f1.zip › Research_Supplementary_revision.docx]

Supplementary Information for

**A Humidity-powered Soft Robot with Fast Rolling Locomotion**

Lei Fu^1,2§^, Weiqiang Zhao^3,4§^, Jiayao Ma^1,2§^, Mingyuan Yang^1,2^, Xinmeng Liu^3,4^, Lei Zhang^3,4*^, Yan Chen^1,2*^

^1^School of Mechanical Engineering, Tianjin University, Tianjin, 300350, China

^2^Key Laboratory of Mechanism Theory and Equipment Design of Ministry of Education, Tianjin University, Tianjin, 300350, China

^3^Department of Biochemical Engineering, School of Chemical Engineering and Technology, Tianjin University, Tianjin, 300350, China

^4^Frontier Science Center for Synthetic Biology and Key Laboratory of Systems Bioengineering (MOE), Tianjin University, Tianjin, 300350, China

^§^Joint first author, ^*^Joint corresponding author

Correspondence should be addressed to Lei Zhang; [lei_zhang@tju.edu.cn](mailto:lei_zhang@tju.edu.cn) and Yan Chen; [yan_chen@tju.edu.cn](mailto:yan_chen@tju.edu.cn)

Note S1. Materials

Agarose powder was purchased from Shanghai Yuanye Bio-Technology Co., Ltd. (Shanghai, China). Phenol red was purchased from Beijing Solarbio Technology Co., Ltd. (Beijing, China). N, N-Dimethylformadide (DMF) was purchased from Tianjin Concord Technology Co., Ltd. (Tianjin, China). Double-sided adhesive tape was purchased from M&G Chenguang Stationery Co., Ltd. (Shanghai, China). Polyethylene terephthalate (PET) sheet was purchased from Tiansheng Plastic Material Co., Ltd. (Guangdong, China). Filter paper was purchased from Hangzhou Special Paper Industry Co., Ltd. (Hangzhou, China).

To demonstrate the capability of the agarose film for continuous motion, we designed the device as shown in Fig. S1(a) to demonstrate the capability of the agarose film for continuous motion. The device was composed of a motor-driven disc which had a 32mm diameter hole covered by filter paper, a vessel filled with water, and a horizontal arm to hold the agarose film. The geometry of the film was *t*=10 μm, *L*=12 mm, *w*=5 mm, the distance from the film to the disc was 3.5 mm, and the humidity on the filter paper surface was about 60%. During experiment, the disc rotated at 8.1 s per cycle. When the filter paper moved underneath the film, it was hydrated by moisture and bent upward, and when the filter paper moved away from the film, it was dehydrated and unbent. As can be shown in Movie S6, the film was stable without the vessel, and then started to bend and unbend when the vessel was in place. In addition, the film returned almost completely to the neutral position after dehydration in the early stage. When it underwent the 568th cycle, it was noticed that there was a slight upward deflection left in the film after dehydration as shown in Fig. S1(b), which gradually increased as the cycle went on. At the 1061th cycle, the ratio of the residual deflection *h*_2_ after dehydration to the maximum deflection *h*_1_ after hydration reached about 40%, at which point we considered the residual deformation might be too large to effectively actuate the robot. Overall, the experimental result indicated that the film could undergo over 1000 cycles without saturation, which made it possible for the Hydrollbot to function for a relatively long period of time. The whole experiment has been recorded in Movie S6.


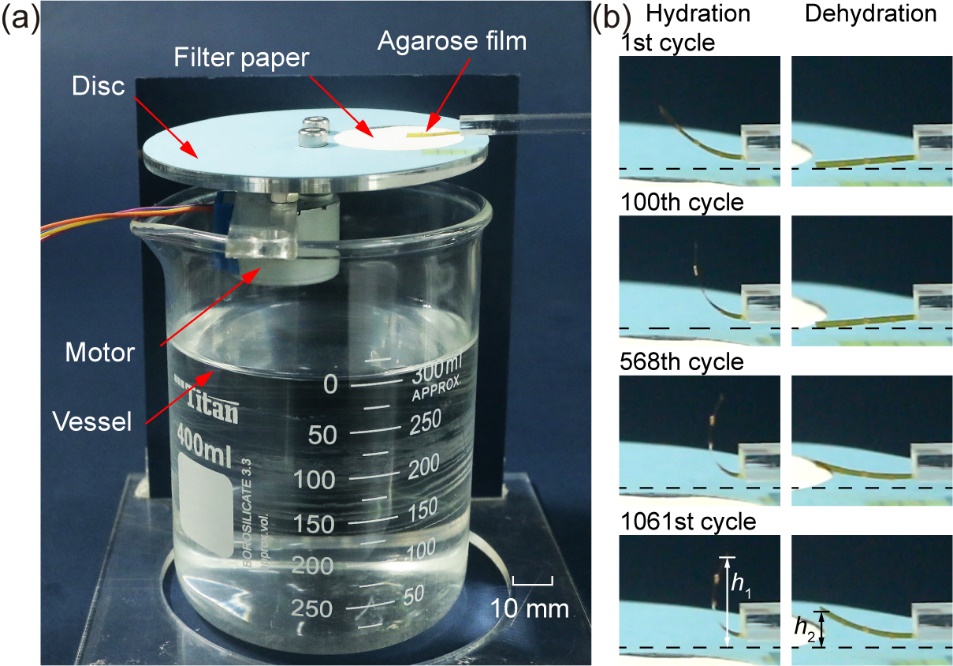


**Figure S1. The cyclic hydration and dehydration of the agarose film. (a)** The experimental setup. **(b)** The configurations of the agarose film after hydration and dehydration in the 1st, 100th, 568th and 1061st cycles.

Note S2. Setup of the locomotion experiments of Hydrollbots

In order to provide a constant environmental humidity for the locomotion experiments of Hydrollbots, an airtight control box with latex gloves was customized. The size of the box was 1600 mm × 1000 mm × 1200 mm (L × W × H), and the desiccant (allochroic silicagel) was used to control the humidity of the box to a constant 20% RH. To achieve locomotion, Hydrollbots with different geometric parameters were placed on wet filter paper (adjustable humidity) above the water in the vessels (Figure S2). The humidity of the filter paper could be adjusted by changing the temperature (23℃ ~ 61℃) of the water. The optimization experiments of geometric parameters and humidity parameters were carried out on a circular filter paper (D = 150 mm) for the accessible records. In order to provide a long enough track for the locomotion of robots, the experiments were carried out on rectangular filter paper (300 mm × 150 mm). Humidity sensor (±2% RH, Anymetre TH21E, China) was placed on the substrate to measure the humidity. The locomotion behavior of Hydrollbot was recorded using a camera (LEICA SUMMARIT-H, Germany).


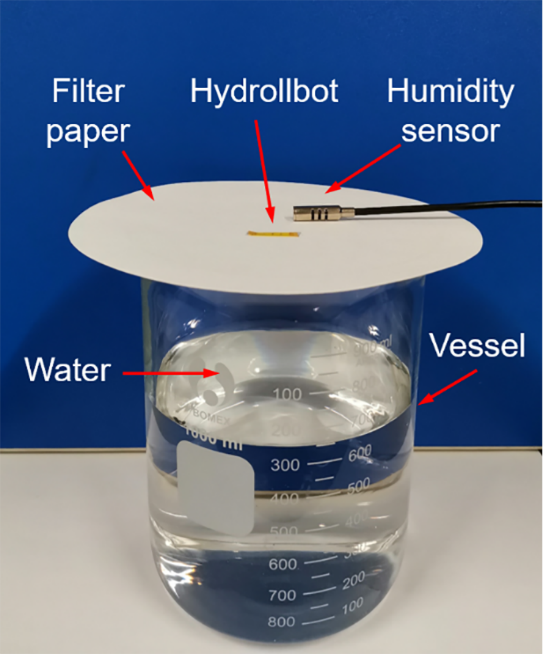


**Figure S2. The experimental setup for the locomotion of Hydrollbots.**

Note S3. Mechanisms to prevent undesirable rolling

When the foot angle or/and film length are not appropriate, undesirable rolling of the Hydrollbot will occur, especially reverse rolling. The probability of reverse rolling can be calculated using the number of reverse rolling and the total rolling number of the Hydrollbot.

The locomotion of the Hydrollbot can be significantly influenced by its foot angle, which may cause reverse rolling. In this work, three foot angles (90°, 120° and 150°) were evaluated. When the foot angle was set to 90°, the Hydrollbot rolled forward 19 cycles and rolled reverse 8 cycles in a 27 cycles experiment. In this case, the reverse rolling probability was ~30%. In comparison, it was found that when the foot angle was set to 120° or 150°, reverse rolling could be avoided. However, the 150° feet tend to hinder forward rolling process, sometimes causing the robots easily got stuck and could not access the recovery state (Figure 2(g)). In comparison, robots with 120° feet can motion most successfully without reverse rolling or any other difficulties. Therefore, 120° was selected as the optimized foot angle for further experiments.

Another important issue is, if the Hydrollbot is too long, reverse rolling can also occur. When *L* = 21 mm and *L*_t_ = 3 mm, due to a large active length (*L*_r_ = *L* – *L*_t_), the Hydrollbot rolled forward 27 cycles and rolled reverse 9 cycles in a 36 cycles experiment. In this case, the probability of reverse rolling was calculated as 25%. However, when *L*_t_ was ≥ 4.5 mm and *L*_r_ was decreased, reverse rolling could be prevented.

Note S4. The lists of data in Figure 3

Date of locomotion velocity of the Hydrollbot with different structural parameters and environmental humidities in Figure 3 are provided in Tables S1-S6. Data of locomotion velocity and body mass of different robots and animals in Figure 3 are provided in Tables S7-S9.

**Table S1. When *L* = 18 mm****,** *L*_t_ **= 3 mm and *RH* ≈ 60%, *v* and T *vs.* *t*.** **(Figure 3(a))**

| ***t* (μm)** | ***T* (s)** | **Error Bar (s)** | ***v* (BL/s)** | **Error Bar (BL/s)** |
| --- | --- | --- | --- | --- |
| 10 | 3.145 | ±0.471 | 0.326 | ±0.056 |
| 15 | 6.553 | ±2.161 | 0.172 | ±0.060 |
| 20 | 13.042 | ±1.767 | 0.078 | ±0.011 |
| 25 | 17.246 | ±4.607 | 0.062 | ±0.014 |
| 30 | 29.067 | ±4.934 | 0.035 | ±0.006 |

**Table S2.** **When *t* = 10 μm,** *L*_t_ **= 3 mm and *RH* ≈ 60%, *v* and *T* *vs. L*. (Figure 3(b))**

| ***L* (mm)** | ***T* (s)** | **Error Bar (s)** | ***v* (BL/s)** | **Error Bar (BL/s)** |
| --- | --- | --- | --- | --- |
| 12 | 9.042 | ±1.848 | 0.115 | ±0.019 |
| 15 | 5.383 | ±1.245 | 0.197 | ±0.048 |
| 18 | 2.894 | ±0.488 | 0.356 | ±0.074 |
| 21 | 3.138 | ±0.696 | 0.338 | ±0.097 |

**Table S3.** **When *t* = 20 μm,** *L*_t_ **= 3 mm and *RH* ≈ 60%, *v* and *T* *vs.* *L*. (Figure 3(c))**

| ***L* (mm)** | | ***T* (s)** | **Error Bar (s)** | ***v* (BL/s)** | **Error Bar (BL/s)** |
| --- | --- | --- | --- | --- | --- |
| 15 | 17.105 | | ±3.497 | 0.061 | ±0.013 |
| 18 | 13.470 | | ±1.640 | 0.075 | ±0.010 |
| 21 | 6.580 | | ±1.705 | 0.162 | ±0.037 |
| 24 | 5.188 | | ±0.841 | 0.198 | ±0.033 |
| 27 | 3.406 | | ±0.588 | 0.303 | ±0.053 |

**Table S4.** **When *t* = 30 μm,** *L*_t_ **= 7 mm and *RH* ≈ 60%, *v* and *T* *vs.* *L*. (Figure 3(d))**

| ***L* (mm)** | | ***T* (s)** | **Error Bar (s)** | ***v* (BL/s)** | **Error Bar (BL/s)** |
| --- | --- | --- | --- | --- | --- |
| 25 | 38.500 | | ±6.062 | 0.027 | ±0.004 |
| 30 | 15.250 | | ±4.710 | 0.071 | ±0.018 |
| 35 | 13.250 | | ±3.110 | 0.081 | ±0.022 |
| 40 | 7.600 | | ±2.107 | 0.142 | ±0.039 |
| 45 | 8.714 | | ±1.485 | 0.119 | ±0.023 |

**Table S5.** **When *t* = 20 μm, *L* = 18 mm and *RH* ≈ 60%, *v* and *T* *vs.*** *L*_t_***/L*. (Figure 3(e))**

| *L*_t_***/L*** | ***T* (s)** | **Error Bar (s)** | ***v* (BL/s)** | **Error Bar (BL/s)** |
| --- | --- | --- | --- | --- |
| 0.139 | 13.295 | ±2.879 | 0.080 | ±0.024 |
| 0.167 | 13.470 | ±1.640 | 0.075 | ±0.010 |
| 0.194 | 14.500 | ±1.783 | 0.070 | ±0.009 |
| 0.222 | 16.925 | ±2.792 | 0.061 | ±0.011 |

**Table S6.** **When *t* = 10 μm, *L* = 18 mm and** *L*_t_ **= 3 mm, *v* and *T* *vs.* *RH.* (Figure 3(f))**

| ***RH* (%)** | ***T* (s)** | **Error Bar (s)** | ***v* (BL/s)** | **Error Bar (BL/s)** |
| --- | --- | --- | --- | --- |
| 40 | 18.390 | ±3.032 | 0.056 | ±0.010 |
| 45 | 13.450 | ±3.575 | 0.080 | ±0.021 |
| 50 | 7.820 | ±1.849 | 0.135 | ±0.030 |
| 55 | 4.768 | ±0.947 | 0.219 | ±0.048 |
| 60 | 3.281 | ±0.799 | 0.323 | ±0.079 |
| 65 | 2.361 | ±1.006 | 0.505 | ±0.070 |
| 70 | 1.435 | ±0.227 | 0.714 | ±0.047 |

**Table S7. Locomotion velocity and body mass of soft robots actuated by constant environment. (Figure 3(h))**

| **Label or name of soft robot** | **Drive type** | **Body mass (g)** | **Maximum velocity (BL/s)** | **References** |
| --- | --- | --- | --- | --- |
| HD^22^ | Humidity-driven | 0.039 | 0.0038 | *(22)* |
| TD^32^ | Thermo-driven | 1.67 | 0.126 | *(32)* |
| Hydrollbot | Humidity-driven | 0.0068 | 0.714 | This work |

**Table S8. Locomotion velocity and body mass of soft robots actuated by various environment. (Figure 3(h))**

| **Label of soft robot** | **Drive type** | **Body mass (g)** | **Maximum velocity (BL/s)** | **References** |
| --- | --- | --- | --- | --- |
| LD^1^ | Light-driven | 0.0097 | 0.125 | *(1)* |
| LD^2^ | Light-driven | 0.00003 | 0.2 | *(2)* |
| HD^22^ | Humidity-driven | 0.035 | 0.24 | *(22)* |
| LD^44^ | Light-driven | 0.0071 | 0.023 | *(44)* |
| HD^45^ | Humidity-driven | 0.0074 | 0.084 | *(45)* |
| HD^46^ | Humidity-driven | 0.02 | 0.024 | *(46)* |
| LD^47^ | Light-driven | 0.11 | 0.019 | *(47)* |

**Table S9. Locomotion velocity and body mass of soft robot actuated by external device. (Figure 3(h))**

| **Label of soft robot** | **Drive type** | **Body mass (g)** | **Maximum velocity (BL/s)** | **References** |
| --- | --- | --- | --- | --- |
| ED^48^ | Electro-driven | 3 | 0.56 | *(48)* |
| ED^49^ | Electro-driven | 4.4 | 0.25 | *(49)* |
| ED^50^ | Electro-driven | 19 | 0.105 | *(50)* |
| ED^51^ | Electro-driven | 26 | 0.1 | *(51)* |
| PD^52^ | Pneumatic-driven | 40 | 0.027 | *(52)* |
| PD^53^ | Pneumatic-driven | 63 | 0.039 | *(53)* |
| ED^54^ | Electro-driven | 126 | 0.38 | *(54)* |
| PD^55^ | Pneumatic-driven | 135 | 0.05 | *(55)* |

Note S5. Locomotion speed of Hydrollbots with different structural parameters.

To build an analytical model to estimate the locomotion speed of Hydrollbots, the following assumptions were made. First of all, during locomotion the robot absorbed humidity energy from the environment at a constant rate, which was used to bend the robot and overcome its gravity. Second, water desorption rate was ignored since it was much slower than water absorption rate. In other words, the robot did not lose humidity energy during motion. Then we utilized energy method to derive the time required for each locomotion stage in Figures 2(a)-2(f) of the main paper, which was redrawn in Figure S3 with more detailed parameters.

The time for stage 1 from the initial state in Figure S3(a) to the bending state I in Figure S3 (b) was first calculated. According to experimental results, the geometry of the robot in Figure S3(b) could be approximately divided into straight line PM, arch MJ, and straight lines NJ, QN. According to statistics of experimental data, the averaged central angle of arch MJ, , was $0.956\pi$, and the averaged length of NJ, $u$, was 0.06*L*.

At stage 1, the work done to bend the robot, caused by the change in curvature of arch MJ, was [S1]:


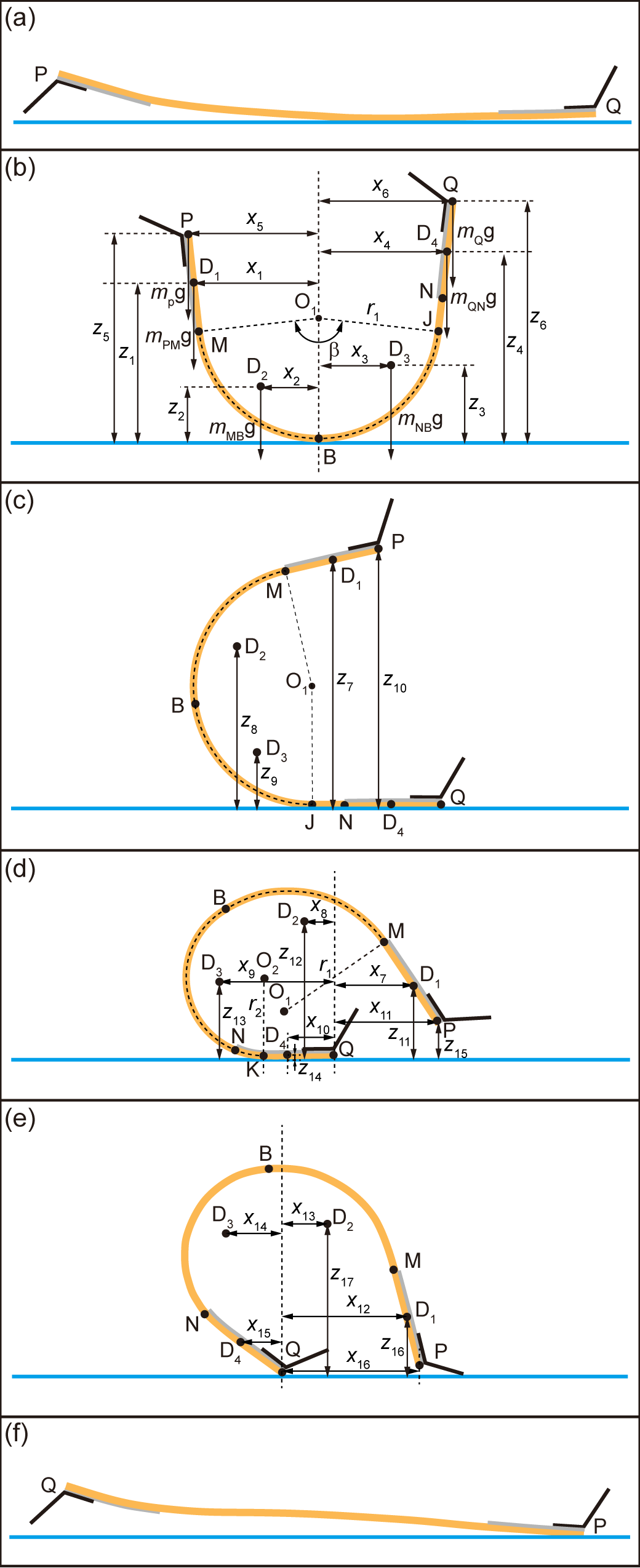


**Figure S3. The analytical model of the** **rolling process of the Hydrollbot.**

|  | $W_{b1}=\frac{EL_{k}w}{2}\int_{-\frac{t}{2}}^{\frac{t}{2}} {(\frac{h}{r_{1}})}^{2}dh=\frac{EL_{k}wt^{3}}{24r_{1}^{2}}$ | (S1) |
| --- | --- | --- |

where $E$ was the elastic modulus of the film, $L_{k}=L-2L_{t}-u$ was arch length of MJ, and $r_{1}=L_{k}/$ was radius of curvature.

Meanwhile, the gravitational potential energy of the robot was also increased at this stage. The total mass of the robot was the summation of segments PM, MB, NB, QN, and the two feet P and Q. The mass of the four segments were respectively:

|  | $m_{\mathrm{PM}}{=m}_{\mathrm{QN}}=\frac{(m_{s}n+{}_{f}wtL)L_{t}}{L}+{}_{t}wt'L_{t}$ |  |
| --- | --- | --- |
|  | $m_{\mathrm{MB}}=\frac{(0.5L-L_{t}-0.5u)(m_{s}n+{}_{f}wtL)}{L}$ | (S2) |
|  | $m_{\mathrm{NB}}=\frac{(0.5L-L_{t}+0.5u)(m_{s}n+{}_{f}wtL)}{L}$ |  |

where $m_{s}$ was the weight of one PET strip, $n$ was the number of the PET strips, ${}_{f}$ was the density of film, $w$ was the width of film, ${}_{t}$ was the density of tape, $t'$ was the thickness of tape.

Note that the PET strips were also considered in the calculation of mass. Since they were evenly distributed on the film, we averaged its mass along the length of the film for simplicity.

Based on the geometry the robot, the centers of gravity of the six parts, D_1_ to D_6_ could be obtained as follows:

|  | | | $x_{1}=r_{1}sin\frac{}{2}+\frac{L_{t}}{2}cos\frac{}{2}$ | |  | |
| --- | --- | --- | --- | --- | --- | --- |
|  | | | $x_{2}=\frac{4r_{1}sin\frac{}{4}}{}sin\frac{}{4}$ | |  | |
|  | | $x_{3}=\frac{L-2L_{t}-u}{L-2L_{t}+u}\frac{4r_{1}sin\frac{}{4}}{}sin\frac{}{4}+\frac{2u}{L-2L_{t}+u}(r_{1}sin\frac{}{2}+\frac{u}{2}cos\frac{}{2})$ | | | |  |
|  | | | $x_{4}=r_{1}sin\frac{}{2}+(\frac{L_{t}}{2}+u)cos\frac{}{2}$ | |  | |
|  | | | $x_{5}=r_{1}sin\frac{}{2}+L_{t}cos\frac{}{2}$ | |  | |
|  | | | $x_{6}=r_{1}sin\frac{}{2}+(L_{t}+u)cos\frac{}{2}$ | |  | |
|  | | | $z_{1}=r_{1}-r_{1}cos\frac{}{2}+\frac{L_{t}}{2}sin\frac{}{2}$ | |  | |
|  | | | $z_{2}=r_{1}-\frac{4r_{1}sin\frac{}{4}}{}cos\frac{}{4}$ | |  | |
|  | $z_{3}=\frac{L-2L_{t}-u}{L-2L_{t}+u}(r_{1}-\frac{4r_{1}sin\frac{}{4}}{}cos\frac{}{4})+\frac{2u}{L-2L_{t}+u}(r_{1}+\frac{u}{2}sin\frac{}{2}$ | | | | |  |
|  |  | | | $-r_{1}cos\frac{}{2})$ | |  |
|  | | | $z_{4}=r_{1}-r_{1}cos\frac{}{2}+(\frac{L_{t}}{2}+u)sin\frac{}{2}$ | |  | |
|  | | | $z_{5}=r_{1}-r_{1}cos\frac{}{2}+L_{t}sin\frac{}{2}$ | |  | |
|  | | | $z_{6}=r_{1}-r_{1}cos\frac{}{2}+(L_{t}+u)sin\frac{}{2}$ | | (S3) | |

Then the gravitational potential energy gained by the robot was

| $W_{g1}=m_{\mathrm{PM}}gz_{1}+m_{\mathrm{MB}}gz_{2}+m_{\mathrm{NB}}gz_{3}+m_{\mathrm{QN}}gz_{4}+m_{p}gz_{5}+m_{q}gz_{6}$ | (S4) |
| --- | --- |

According to the assumptions, the humidity energy absorbed by the robot at stage 1 was

|  | ${W_{h1}=\xi T}_{1}$ | (S5) |
| --- | --- | --- |

where $\xi$ was humidity energy absorption rate, $T_{1}$ was the time for stage 1.

The absorbed humidity energy should be equal to the gains in bending energy and gravitational potential energy, therefore

|  | $W_{h1}=W_{b1}+W_{g1}$ | (S6) |
| --- | --- | --- |

Substituting Equation (S6) into Equation (S5),

|  | $T_{1}=\frac{W_{b1}+W_{g1}}{\xi}$ | (S7) |
| --- | --- | --- |

Since there was currently no theory to determine the humidity energy absorption rate $\xi$, we obtained it through curve fitting. Careful examination of experimental data revealed that this rate was related to ambient humidity, film thickness and length, based on which we assumed the following Equation for the rate.

|  | $\xi=ke^{aRH}t^{0.5}L^{1.5}$ | (S8) |
| --- | --- | --- |

where $k$ and $a$ were two constants.

By fitting Equation (S8) with the experimental data using the least square methods, the two constants were respectively obtained as $k=2.212*{10}^{-4} kg\cdot s^{-3}$ and $a=9.994$, which represents the material property of the agarose film. The curve fitting results using those two values are presented in Figure S4.

During stage 2 from the bending state I in Figure S3(b) to the rolling state I in Figure S3(c), driven by the unbalanced gravitational forces, the robot rolled quickly by $/2$, accounting for only about 4% of the locomotion period on average based on statistics. To estimate the time at this stage, we first calculated the angular acceleration $\alpha_{1}$ about the contact point B.

|  | $I_{1}=m_{\mathrm{PM}}\left( x_{1}^{2}+z_{1}^{2} \right)+m_{\mathrm{MB}}\left( x_{2}^{2}+z_{2}^{2} \right)+m_{\mathrm{NB}}\left( x_{3}^{2}+z_{3}^{2} \right)+m_{\mathrm{QN}}\left( x_{4}^{2}+z_{4}^{2} \right)$ | | | | |  | |
| --- | --- | --- | --- | --- | --- | --- | --- |
|  |  | | $+m_{P}\left( x_{5}^{2}+z_{5}^{2} \right)+m_{Q}\left( x_{6}^{2}+z_{6}^{2} \right)$ | | | (S9) | |
|  | | $M_{1}=-m_{\mathrm{PM}}gx_{1}-m_{\mathrm{MB}}gx_{2}+m_{\mathrm{NB}}gx_{3}+m_{\mathrm{QN}}gx_{4}-m_{p}gx_{5}+m_{q}gx_{6}$ | | | | | (S10) |
|  | | | | $\alpha_{1}=\frac{M_{1}}{I_{1}}$ | (S11) | | |

where $I_{1}$ was the moments of inertia in Figure S3(b),$M_{1}$ was the moment applied to the robot.


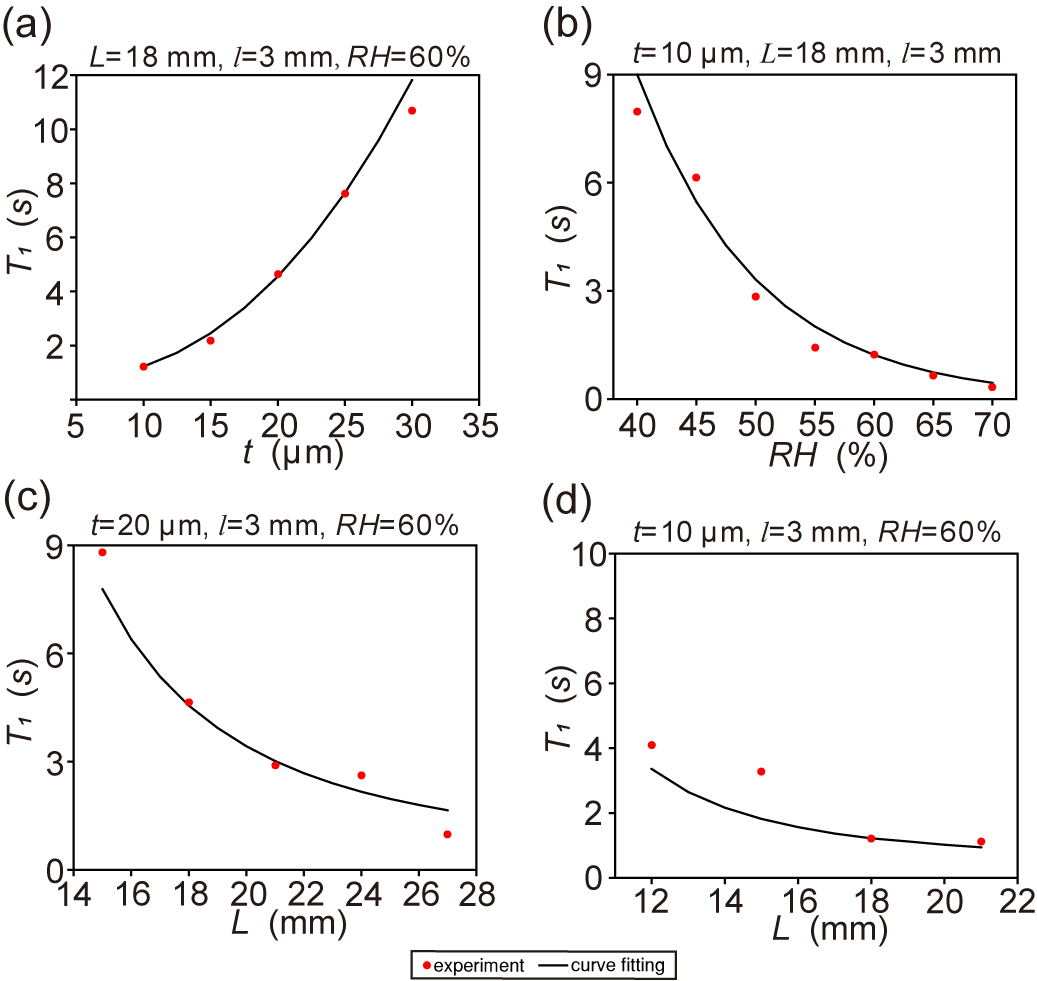


**Figure S4.** **The** **curve fitting result of** **the time** $\boldsymbol{T}_{\mathbf{1}}$ **in four different conditions with** **energy absorption rate** $\boldsymbol{\xi}$**.**

During rolling, the contact point continuously changed, and the angular acceleration also reduced until it reached zero. Since the rolling time was relatively short in comparison with the locomotion period, we simplified the calculation by considering a constant angular acceleration as half of the initial value, and obtained the time of stage 2 as follows

|  | $T_{2}=\sqrt{\frac{2}{\alpha_{1}}}$ | (S12) |
| --- | --- | --- |

During stage 3 from the rolling state I in Figure S3(c) to the bending state II in Figure S3(d), it was observed that segments BJ, NJ, and a portion of segment QN, referred to as KN, were bent together to a radius of curvature $r_{2}$, whereas segments PM and MB kept their shapes. This bending process continued until the center of gravity of the robot passed point Q in the horizontal direction, when the robot was unbalanced and rolled again. This could be judged as

| $m_{\mathrm{PM}}x_{7}+m_{\mathrm{MB}}x_{8}-m_{\mathrm{NB}}x_{9}-m_{\mathrm{QN}}x_{10}+m_{p}x_{11}=0$ | (S13) |
| --- | --- |

in which

|  | | $x_{7}=\frac{L_{t}}{2}cos(\frac{L-2L_{t}-u}{2r_{1}}+\frac{L+u-2(L_{t}-l_{\mathrm{KN}})}{{2r}_{2}}-\pi)+(r_{1}-r_{2})sin(\pi$ | | | | | | |  |
| --- | --- | --- | --- | --- | --- | --- | --- | --- | --- |
|  | |  | | | $\boldsymbol{-}\frac{\boldsymbol{L+u-2}\left( \boldsymbol{L}_{\boldsymbol{t}}\boldsymbol{-}\boldsymbol{l}_{\mathbf{KN}} \right)}{\boldsymbol{2r}_{\boldsymbol{2}}}\boldsymbol{)+}\boldsymbol{r}_{\boldsymbol{1}}\mathbf{sin}\boldsymbol{(}\frac{\boldsymbol{L-2}\boldsymbol{L}_{\boldsymbol{t}}\boldsymbol{-u}}{\boldsymbol{2}\boldsymbol{r}_{\boldsymbol{1}}}\boldsymbol{+}\frac{\boldsymbol{L+u-2(}\boldsymbol{L}_{\boldsymbol{t}}\boldsymbol{-}\boldsymbol{l}_{\mathbf{KN}}\boldsymbol{)}}{\boldsymbol{2r}_{\boldsymbol{2}}}$ | | | | |
|  | |  | | | $-\pi)-L_{t}+l_{\mathrm{KN}}$ | | | |  |
|  | $\boldsymbol{x}_{\boldsymbol{8}}\boldsymbol{=-}\frac{\boldsymbol{4r}_{\boldsymbol{1}}^{\boldsymbol{2}}\boldsymbol{sin}\frac{\boldsymbol{L-2}\boldsymbol{L}_{\boldsymbol{t}}\boldsymbol{-u}}{\boldsymbol{4}\boldsymbol{r}_{\boldsymbol{1}}}}{\boldsymbol{L-2}\boldsymbol{L}_{\boldsymbol{t}}\boldsymbol{-u}}\boldsymbol{sin}\left( \boldsymbol{\pi-}\frac{\boldsymbol{L+u-2}\boldsymbol{L}_{\boldsymbol{t}}\boldsymbol{+2}\boldsymbol{l}_{\mathbf{KN}}}{\boldsymbol{2r}_{\boldsymbol{2}}}\boldsymbol{-}\frac{\boldsymbol{L-2}\boldsymbol{L}_{\boldsymbol{t}}\boldsymbol{-u}}{\boldsymbol{4}\boldsymbol{r}_{\boldsymbol{1}}} \right)\boldsymbol{-}\boldsymbol{L}_{\boldsymbol{t}}$ | | | | | | | | |
|  |  | | $+l_{\mathrm{KN}}+\left( r_{1}-r_{2} \right)\sin\left( \pi-\frac{L+u-2L_{t}+2l_{\mathrm{KN}}}{{2r}_{2}} \right)$ | | | | |  | |
|  | | | | | | $\boldsymbol{x}_{\boldsymbol{9}}\boldsymbol{=}\frac{\boldsymbol{4r}_{\boldsymbol{2}}^{\boldsymbol{2}}\boldsymbol{sin}\frac{\boldsymbol{L-2}\boldsymbol{L}_{\boldsymbol{t}}\boldsymbol{+u}}{\boldsymbol{4r}_{\boldsymbol{2}}}}{\boldsymbol{L-2}\boldsymbol{L}_{\boldsymbol{t}}\boldsymbol{+u}}\boldsymbol{sin}\left( \frac{\boldsymbol{L-2}\boldsymbol{L}_{\boldsymbol{t}}\boldsymbol{+u}}{\boldsymbol{4r}_{\boldsymbol{2}}}\boldsymbol{+}\frac{\boldsymbol{l}_{\mathbf{KN}}}{\boldsymbol{r}_{\boldsymbol{2}}} \right)\boldsymbol{+}\boldsymbol{L}_{\boldsymbol{t}}\boldsymbol{-}\boldsymbol{l}_{\mathbf{KN}}$ | | | |
|  | | | | | | $x_{10}=L_{t}-l_{\mathrm{KN}}+\frac{{2r}_{2}^{2}sin\frac{l_{\mathrm{KN}}}{{2r}_{2}}}{L_{t}}sin\frac{l_{\mathrm{KN}}}{{2r}_{2}}-\frac{{(L_{t}-l_{\mathrm{KN}})}^{2}}{2L_{t}}$ |  | | |
|  | $x_{11}=L_{t}cos(\frac{L-2L_{t}-u}{2r_{1}}+\frac{L+u-2(L_{t}-l_{\mathrm{KN}})}{{2r}_{2}}-\pi)+(r_{1}-r_{2})sin(\pi$ | | | | | | |  | |
|  |  | | | $\boldsymbol{-}\frac{\boldsymbol{L+u-2}\left( \boldsymbol{L}_{\boldsymbol{t}}\boldsymbol{-}\boldsymbol{l}_{\mathbf{KN}} \right)}{\boldsymbol{2r}_{\boldsymbol{2}}}\boldsymbol{)+}\boldsymbol{r}_{\boldsymbol{1}}\mathbf{sin}\boldsymbol{(}\frac{\boldsymbol{L-2}\boldsymbol{L}_{\boldsymbol{t}}\boldsymbol{-u}}{\boldsymbol{2}\boldsymbol{r}_{\boldsymbol{1}}}\boldsymbol{+}\frac{\boldsymbol{L+u-2(}\boldsymbol{L}_{\boldsymbol{t}}\boldsymbol{-}\boldsymbol{l}_{\mathbf{KN}}\boldsymbol{)}}{\boldsymbol{2r}_{\boldsymbol{2}}}$ | | | | | |
|  |  | | | $-\pi)-L_{t}+l_{\mathrm{KN}}$ | | | |  | |

To solve $r_{2}$ and the length of KN, $l_{\mathrm{KN}}$, we first assumed $l_{\mathrm{KN}}=0$, and continuously reduced $r_{2}$. As $r_{2}$ was reduced, the configuration of the robot changed, leading to variation in center of gravity. If the center of gravity passed point Q before point P touched the moist surface, the value of $r_{2}$ was adopted. If not, we slightly increased $l_{\mathrm{KN}}$ and did the calculation again, until we found the combination of $r_{2}$ and $l_{\mathrm{KN}}$ that caused the center of gravity passed Q before point P touched the moist surface.

With $r_{2}$ and $l_{\mathrm{KN}}$, the configuration of the robot in Figure S3(d) could be obtained, and the work done to further bend the robot, caused by the change in curvature of BJ, NJ, and QN, was

| $W_{b2}=\frac{E\left( 0.5L-L_{t}-0.5u \right)wt^{3}}{24r_{2}^{2}}-\frac{E\left( 0.5L-L_{t}-0.5u \right)wt^{3}}{24r_{1}^{2}}+\frac{E\left( l_{\mathrm{KN}}+u \right)wt^{3}}{24r_{2}^{2}}$ | (S14) |
| --- | --- |

The variation in gravitational potential energy at stage 3 was

|  | | $z_{7}=r_{1}+r_{1}sin(\frac{L-2L_{t}-u}{r_{1}}-\frac{\pi}{2})+\frac{L_{t}}{2}sin(\pi-\frac{L-2L_{t}-u}{r_{1}})$ | | | | |  | | | | |
| --- | --- | --- | --- | --- | --- | --- | --- | --- | --- | --- | --- |
|  | | $z_{8}=r_{1}+\frac{{4r}_{1}^{2}sin\frac{L-2L_{t}-u}{4r_{1}}sin(\frac{3L-6L_{t}-3u}{4r_{1}}-\frac{\pi}{2})}{L-2L_{t}-u}$ | | | | |  | | | | |
|  | | $z_{9}=\frac{\left( L-2L_{t}-u \right)r_{1}-{4r}_{1}^{2}sin\frac{L-2L_{t}-u}{4r_{1}}cos\frac{L-2L_{t}-u}{4r_{1}}}{L-2L_{t}+u}$ | | | | |  | | | | |
|  | | $z_{10}=r_{1}+r_{1}sin(\frac{L-2L_{t}-u}{r_{1}}-\frac{\pi}{2})+L_{t}sin(\pi-\frac{L-2L_{t}-u}{r_{1}})$ | | | | |  | | | | |
|  | $z_{11}=r_{2}+r_{1}cos(\frac{L-2L_{t}-u}{2r_{1}}+\frac{L+u-2(L_{t}-l_{\mathrm{KN}})}{{2r}_{2}}-\pi)-(r_{1}-r_{2})$ | | | | | | | |  |  |  |
|  |  | | | | $cos\left( \pi-\frac{L+u-2(L_{t}-l_{\mathrm{KN}})}{{2r}_{2}} \right)-\frac{L_{t}}{2}sin(\frac{L-2L_{t}-u}{2r_{1}}$ | | | |  | | |
|  |  | | | | $+\frac{L+u-2(L_{t}-l_{\mathrm{KN}})}{{2r}_{2}}-\pi)$ | | | |  | | |
|  | $z_{12}=r_{2}+\frac{{4r}_{1}^{2}sin\frac{L-2L_{t}-u}{4r_{1}}cos\left( \pi-\frac{L+u-2L_{t}+2l_{\mathrm{KN}}}{{2r}_{2}}-\frac{L-2L_{t}-u}{4r_{1}} \right)}{L-2L_{t}-u}$ | | | | | | | |  | | |
|  |  | | | $-(r_{1}-r_{2})cos\left( \pi-\frac{L+u-2L_{t}+2l_{\mathrm{KN}}}{{2r}_{2}} \right)$ | | | | |  | | |
|  | $z_{13}=r_{2}-\frac{{4r}_{2}^{2}sin\frac{L-2L_{t}+u}{{4r}_{2}}cos(\frac{L-2L_{t}+u}{{4r}_{2}}+\frac{l_{\mathrm{KN}}}{r_{2}})}{L-2L_{t}+u}$ | | | | | | | |  | |  |
|  | $z_{14}=\frac{l_{\mathrm{KN}}}{L_{t}}(r_{2}-\frac{{2r}_{2}^{2}sin\frac{l_{\mathrm{KN}}}{{2r}_{2}}}{l_{\mathrm{KN}}}cos\frac{l_{\mathrm{KN}}}{{2r}_{2}})$ | | | | | | | |  | |  |
|  | $z_{15}=r_{2}+r_{1}cos(\frac{L-2L_{t}-u}{2r_{1}}+\frac{L+u-2(L_{t}-l_{\mathrm{KN}})}{{2r}_{2}}-\pi)-(r_{1}-r_{2})$ | | | | | | | | |  | |
|  |  | | | | | $cos\left( \pi-\frac{L+u-2(L_{t}-l_{\mathrm{KN}})}{{2r}_{2}} \right)-L_{t}sin(\frac{L-2L_{t}-u}{2r_{1}}$ | | | |  | |
|  |  | | | | | $+\frac{L+u-2(L_{t}-l_{\mathrm{KN}})}{{2r}_{2}}-\pi)$ | | | |  | |
|  | $W_{g2}=m_{\mathrm{PM}}gz_{11}+m_{\mathrm{MB}}gz_{12}+m_{\mathrm{NB}}gz_{13}+m_{\mathrm{QN}}gz_{14}+m_{p}gz_{15}-(m_{\mathrm{PM}}gz_{7}$ | | | | | | |  | | |  |
|  |  | | $+m_{\mathrm{MB}}gz_{8}+m_{\mathrm{NB}}gz_{9`}+m_{p}gz_{10})$ | | | | | (S15) | | |  |

Therefore, the time for stage 3 was

|  | $T_{3}=\frac{W_{b2}+W_{g2}}{\xi}$ | (S16) |
| --- | --- | --- |

During stage 4 from the bending state II in Figure S3(d) to the rolling state II in Figure S3(e), the robot rolled again due to gravity. Through the statistics of experimental data, the time of this process accounted for 2% of the locomotion period on average. Using the same simplification, the time of stage 4 could be calculated as.

|  | | | $x_{12}=\sqrt{x_{7}^{2}+z_{11}^{2}}cos(arctan\frac{z_{11}}{x_{7}}-arctan\frac{z_{15}}{x_{11}})$ | |  | | |
| --- | --- | --- | --- | --- | --- | --- | --- |
|  | | | $x_{13}=\sqrt{x_{8}^{2}+z_{12}^{2}}\vert cos\left( arctan\frac{z_{12}}{x_{8}}-arctan\frac{z_{15}}{x_{11}} \right)\vert$ | |  | | |
|  | | | $x_{14}=\sqrt{x_{9}^{2}+z_{13}^{2}}cos(arctan\frac{z_{13}}{x_{9}}+arctan\frac{z_{15}}{x_{11}})$ | |  | | |
|  | | | $x_{15}=\sqrt{x_{10}^{2}+z_{14}^{2}}cos(arctan\frac{z_{14}}{x_{10}}+arctan\frac{z_{15}}{x_{11}})$ | |  | | |
|  | | | $x_{16}=\sqrt{x_{11}^{2}+z_{15}^{2}}$ | |  | | |
|  | | | $z_{16}=\sqrt{x_{7}^{2}+z_{11}^{2}}sin\left( arctan\frac{z_{11}}{x_{7}}-arctan\frac{z_{15}}{x_{11}} \right)$ | |  | | |
|  | | | $z_{17}=\sqrt{x_{8}^{2}+z_{12}^{2}}\vert sin\left( arctan\frac{z_{12}}{x_{8}}-arctan\frac{z_{15}}{x_{11}} \right)\vert$ | |  | | |
|  | $I_{2}=m_{PM}\left( x_{7}^{2}+z_{11}^{2} \right)+m_{MB}\left( x_{8}^{2}+z_{12}^{2} \right)+m_{NB}\left( x_{9}^{2}+z_{13}^{2} \right)$ | | | | |  | |
|  |  | | | $+m_{QN}\left( x_{10}^{2}+z_{14}^{2} \right)+m_{P}\left( x_{11}^{2}+z_{15}^{2} \right)$ | |  | |
|  | | $M_{2}=m_{\mathrm{PM}}gx_{12}+m_{\mathrm{MB}}gx_{13}-m_{\mathrm{NB}}gx_{14}-m_{\mathrm{QN}}gx_{15}+m_{p}gx_{16}$ | | | | |  |
|  | | | $\alpha_{2}=\frac{M_{2}}{I_{2}}$ | |  | | |
|  | | | $T_{4}=\sqrt{\frac{4arctan\frac{z_{15}}{x_{11}}}{\alpha_{2}}}$ | | (S17) | | |

Finally was stage 5 from the bending state II in Figure S3(e) to the recovery state in Figure S3(f), During this stage, the robot absorbed water to unbend to the straight state. It was observed from experiment that at the beginning of this stage, segment QB quickly bounced back, leading to a noticeable reduction in curvature, see Figure S5. Such a quick change in shape could not be caused by water absorption, but most likely by release of elastic energy stored in the segment. Segment PB, on the contrary, showed no such bounce. Afterward, the robot was steadily straightened to flat.


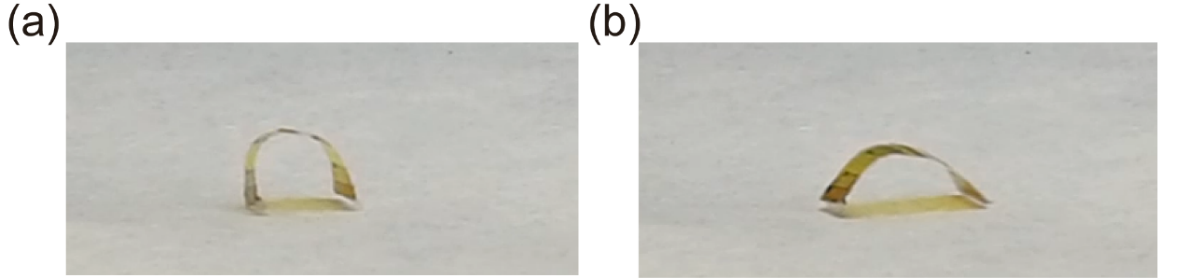


**Figure S5. The states before (a) and after (b) bouncing** **of the Hydrollbot** (film length *L* = 18 mm, film thickness *t* = 10 μm, tape length *L*_t_ = 3 mm, humidity *RH* ≈ 70%).

Since the elastic energy release of segment QB was difficult to quantify, we estimated the time at this stage by considering only PB. The work done to bend the robot, caused by the change in curvature of MB, was

|  | $W_{b3}=\frac{E(0.5L-L_{t}-0.5u)wt^{3}}{24r_{1}^{2}}$ | (S18) |
| --- | --- | --- |

The variation in gravitational potential energy was

|  | $W_{g3}=-m_{\mathrm{PM}}gz_{16}-m_{\mathrm{MB}}gz_{17}$ | (S19) |
| --- | --- | --- |

Then the time for stage 5 could be obtained as

|  | $T_{5}=\frac{W_{b3}+W_{g3}}{{0.47}^{1.5}\xi}$ | (S20) |
| --- | --- | --- |

Note that there was a coefficient ${0.47}^{1.5}$ for $\xi$ since PB considered in the analysis at this stage was 47% of the total length of the robot.

With the time for each stage being obtained, the locomotion period of the robot could be determined

|  | $T=T_{1}+T_{2}+T_{3}+T_{4}+T_{5}$ | (S21) |
| --- | --- | --- |

In order to directly observe and compare the effect of structural parameters on the locomotion of the Hydrollbot, two races of Hydrollbots with different structure parameters were performed.

In the first race, Hydrollbot Ⅰ moved ~7 cm after four rolling cycles, whereas Hydrollbot Ⅱ moved only ~4 cm after two rolling cycles (Figure 3(g)). The average rolling cycle duration and speed are 1.4 s and 0.7 BL/s for Hydrollbot Ⅰ, and 2.8 s and 0.35 BL/s for Hydrollbot ⅠI, respectively. The corresponding video is shown in Movie S3.

In the second race, Hydrollbot Ⅲ moved ~6 cm after four rolling cycles, whereas Hydrollbot Ⅳ moved only 1.2 cm after one rolling cycle (Figure S6). The average rolling cycle duration and speed are 2.5 s and 0.4 BL/s for Hydrollbot ⅠII, and 10 s and 0.1 BL/s for for Hydrollbot IV, respectively.


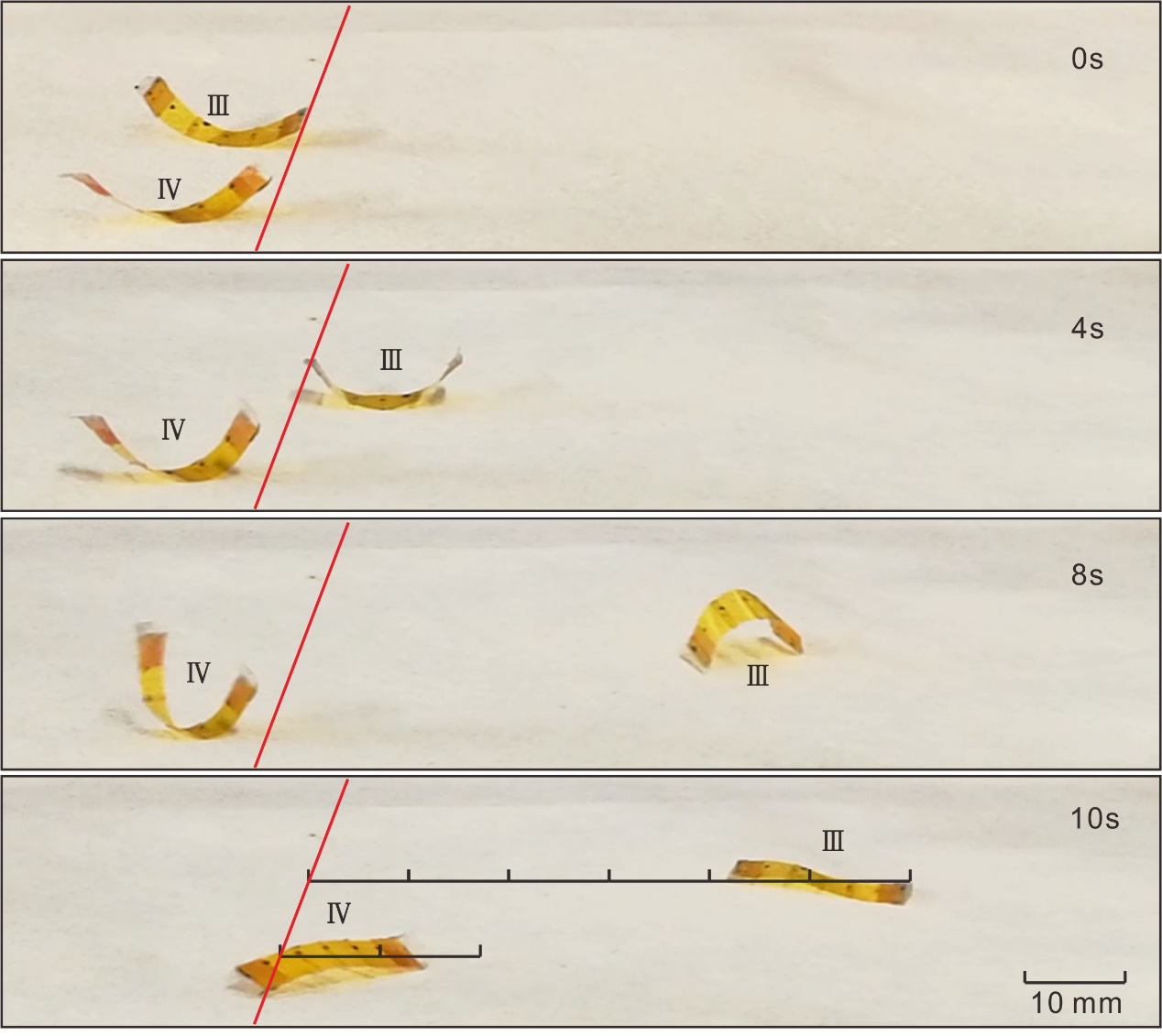


**Figure S6. The rolling comparison of two Hydrollbots with different structure parameters.** Locomotion speed comparison of Hydrollbot Ⅲ (*L* = 18 mm, *t* = 10 μm, and *L*_t_ = 3 mm) and Hydrollbot Ⅳ (*L* = 18 mm, *t* = 20 μm, and *L*_t_ = 3 mm) at *RH* ≈ 60%.

Note S6. The loading carrying capability of the Hydrollbot.

We designed and tested two robots, one without payload and one with payload, to demonstrate the load carrying capability of the Hydrollbot. The geometry of the robots was *t* = 10 μm, *L* = 18 mm, *L*_t_ = 3 mm, and the testing humidity was *RH*≈70%. The robot without payload as shown Fig. S7 (a) had a total weight of 6.2 mg, in which the body (summation of the film 1.8 mg, the two tapes 1.5 mg, and the three PET strips 0.9 mg) was 4.2 mg and the two feet were 2.0 mg. For the one with payload, we added two small pieces of tapes (the blue strips in Fig. S7(b)) with a total weight of 6.2 mg next to the two feet, and therefore the total weight of the robot was doubled to 12.4 mg. The rolling process of the two Hydrollbots is shown in the newly added Movie S4. It can be seen that both robots can roll smoothly, indicating that the proposed Hydrollbot is capable of carrying a payload up to 100% of its own weight.


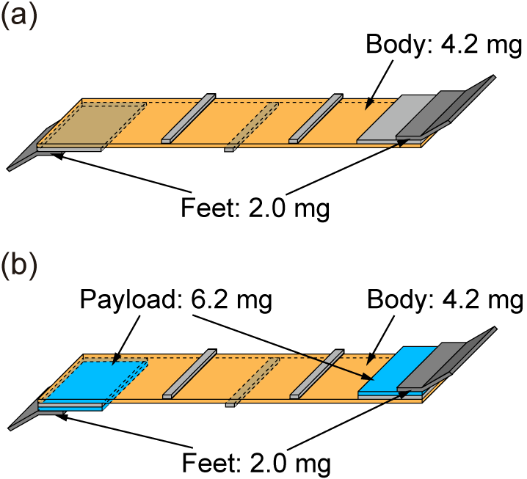


**Figure S7. Design of the Hydrollbots (a) without payload, and (b) with payload.**

**Note 7. The optimization on the width of PET strips and the distance between adjacent strips of the Hydrollbot**

The width of PET strips and the distance between adjacent strips do affect the locomotion speed of the Hydrollbot. For all the robots in this work, the width of the PET strips was 0.5 mm and the pitch (distance) of the strips was 3 mm. To demonstrate the effect of the strips, we designed and tested three robots. The geometry of the robots was *t* = 10 μm, *L* = 18 mm, *L*_t_ = 3 mm, and the testing humidity was *RH*≈70%. We first maintained the pitch of the strips and increased the width from 0.5mm to 1.0 mm. As shown from the rolling process of the robot in Movie S7 and Fig. S8(a), reverse rolling occurred during the locomotion. The same phenomenon was also observed when the width of the strips was kept 0.5 mm and the pitch was reduced from 3 mm to 1.5 mm, see Movie S7 and Fig. S8(b). The reason is that both increasing the width and reducing the pitch lead to an increase in the weight of the robot body. When the robot is at the bending state I as shown in Fig. S8(c), the distance between the mass center of part BP and the contact point B is reduced from $l_{P}$ to $l_{P}'$, and so is the distance between the mass center of part BP and contact point B from $l_{Q}$ to $l_{Q}'$. As a result, the robot is more likely to roll backward, which will in turn severely reduce the locomotion speed. On the other hand, if the pitch of the PET strips is too large, the bending deformation cannot be restricted to along the longitudinal direction of the film, thus leading to uncontrolled bending deformation and locomotion. An example of such a robot with a strip width of 0.5mm and a pitch of 6 mm is shown in Movie S7 and Fig. S8(d).


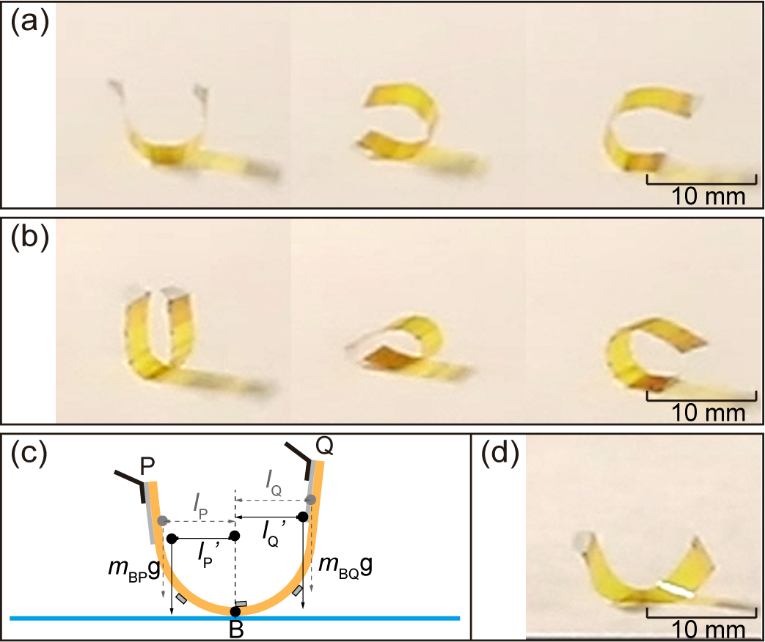


**Figure S8. The effect of the width and pitch of the PET strips on the rolling locomotion.** **(a)** The robot with strip width 1 mm and pitch 3 mm. **(b)** The robot with strip width 0.5 mm and pitch 1.5 mm. **(c)** The change in the mass centers of BQ and BP at the bending state I. **(d)** The robot with strip width 0.5 mm and pitch 6 mm.

**Reference**

[S1] H. S. MARTIN, Elasticity Theory, Applications and Numerics (United States of America: Academic Press, 2005), 104-106.

Supplementary Videos Description

**Movie S1. Preparation of the Hydrollbot**

This video has two parts:

- Preparation of the Hydrollbot.

Firstly, attach tapes to the two ends of the film. Then attach PET strips to different sides of the film alternatively. Lastly, adhere PET feet to the two ends of the film. (Film thickness *t* = 10 μm, film length *L* = 18 mm, tape length *L*_t_ = 3 mm)

- Locomotion states of Hydrollbot in four preparation steps.

(Film thickness *t* = 10 μm, film length *L* = 18 mm, tape length *L*_t_ = 3 mm, humidity *RH* ≈ 60%)

**Movie S2. Rolling process of the Hydrollbot**

The video shows the rolling process of a Hydrollbot (film length *L* = 18 mm, film thickness *t* = 10 μm, tape length *L*_t_ = 3 mm, humidity *RH* ≈ 70%). This robot rolled six cycles in 4.4 s. A four-time slowdown video is to show the detailed rolling process.

**Movie S3. Rolling comparison of Hydrollbots with different structural parameters**

Hydrollbot Ⅰ rolled four cycles (~7 cm) in 5.6 s. Hydrollbot Ⅱ rolled two cycles (~4 cm) in 5.6 s. (Hydrollbot Ⅰ: film length *L* =18 mm, film thickness *t* = 10 μm, tape length *L*_t_ = 3 mm, humidity *RH* ≈ 70%. Hydrollbot Ⅱ: film length *L* = 21mm, film thickness *t* = 20 μm, tape length *L*_t_ =4 mm, humidity *RH* ≈ 70%)

**Movie S4. Rolling process of the Hydrollbots with different weights**

This video has two parts:

- The Hydrollbot without payload (6.2 mg).

The Hydrollbot rolled four cycles in 6.1 s (film thickness *t* = 10 μm, film length *L* = 18 mm, tape length *L*_t_ = 3 mm, humidity *RH* ≈ 70%).

- The Hydrollbot with payload (12.4 mg).

The Hydrollbot rolled four cycles in 4.7 s (film thickness *t* = 10 μm, film length *L* = 18 mm, tape length *L*_t_ = 3 mm, humidity *RH* ≈ 70%).

**Movie S5. Programmable trajectories of the Hydrollbots**

This video has four parts:

- Rolling process of the Hydrollbot with a zigzag trajectory.

The Hydrollbot rolled five cycles in 8 s, and performed two zigzag trajectories. (Parallelogram robot: Film thickness *t* = 10 μm, *L* = 18 mm, *w* = 8 mm, *α* = 60°, humidity *RH* ≈ 60%)

- Rolling process of the Hydrollbot with a hexagon trajectory.

The Hydrollbot rolled five cycles in 14 s, and performed one hexagon trajectory. (Trapezoid robot: film thickness *t* = 10 μm, *L* = 18 mm, *w* = 8 mm, *α* = 60°, humidity *RH* ≈ 60%)

- Rolling process of the Hydrollbot with a circular trajectory.

The Hydrollbot rolled seven cycles in 27 s, and performed one circular trajectory. (Sectorial robot: film thickness *t* = 10 μm, *R* = 26 mm, *r* = 18 mm, *α* = 50°, humidity *RH* ≈ 60%)

- Rolling process of the Hydrollbot with a flower-shaped trajectory.

The Hydrollbot rolled seven cycles in 46 s, and performed one flower-shaped trajectory. (Sectorial robot: film thickness *t* = 10 μm, *R* = 21 mm, *r* = 11 mm, *d* = 4 mm, *α* = 60°, humidity *RH* ≈ 60%)

**Movie S6. Cyclic hydration and dehydration of the agarose film**

The video shows the film was stable during the first cycle without the vessel, and then started to bend and unbend during the 1st, 100th, 568th and 1061st cycles when the vessel was in place (film thickness *t* = 10 μm, film length *L* = 12 mm, film width *w*=5 mm, humidity *RH* ≈ 60%).

**Movie S7. The effect of the width and pitch of the PET strips on the rolling locomotion**

This video has third parts:

- Rolling process of the Hydrollbot with 1-mm-wide PET strips.

The Hydrollbot rolled four cycles in 38 s (film thickness *t* = 10 μm, film length *L* = 18 mm, tape length *L*_t_ = 3 mm, humidity *RH* ≈ 70%).

- Rolling process of the Hydrollbot with 1.5-mm-pitch PET strips.

The Hydrollbot rolled five cycles in 28 s (film thickness *t* = 10 μm, film length *L* = 18 mm, tape length *L*_t_ = 3 mm, humidity *RH* ≈ 70%).

- Rolling process of the Hydrollbot with 6-mm-pitch PET strips.

(Film thickness *t* = 10 μm, film length *L* = 18 mm, tape length *L*_t_ = 3 mm, humidity *RH* ≈ 70%).
